# 3D Imaging of Optical Modes in Dielectric Photonic Nanocavities with Sub-wavelength Field Confinement

**DOI:** 10.1021/acs.nanolett.5c04226

**Published:** 2025-11-10

**Authors:** Michael S. Seifner, Anne Sofie Darket, Ali N. Babar, Babak Vosoughi Lahijani, Rasmus E. Christiansen, Ole Sigmund, Elizaveta Semenova, Søren Stobbe, Philip T. Kristensen, Shima Kadkhodazadeh

**Affiliations:** † DTU Nanolab, 5205 Technical University of Denmark, Fysikvej 307, 2800 Kongens Lyngby, Denmark; ‡ DTU Electro, 5205 Technical University of Denmark, Ørsteds Plads 343, 2800 Kongens Lyngby, Denmark; § DTU Construct, 5205 Technical University of Denmark, Koppels Allé 404, 2800 Kongens Lyngby, Denmark; ∥ NanoPhoton—Center for Nanophotonics, 5205 Technical University of Denmark, Ørsteds Plads 345A, 2800 Kongens Lyngby, Denmark

**Keywords:** dielectric photonic nanocavity, sub-wavelength field confinement, high-*Q* cavity, electron energy-loss spectroscopy, electron tomography, strong light−matter interaction

## Abstract

Dielectric optical cavities are emerging as viable platforms for efficiently concentrating light within extremely small volumes of sub-wavelength dimensions. This breaks with the notion that only plasmonic nanostructures can achieve this scale of confinement and enables strong light–matter interactions without the losses typically associated with metals. Here, we directly visualize the optical modes of a topology-optimized silicon bowtie nanocavity using multi-orientation electron energy-loss spectroscopy. Tomographic reconstruction of the resulting data sets reveals the three-dimensional profiles of several polarized optical modes in close agreement with simulations. A resonance near the telecom wavelength (∼1550 nm) is shown to be tightly localized at the bowtie bridge, confirming its deep sub-wavelength mode volume. These findings establish electron beam spectroscopy as a powerful tool for mapping three-dimensional field confinement in dielectric photonic cavities with potential applications in future photonic and quantum technologies.

## Graphical abstract

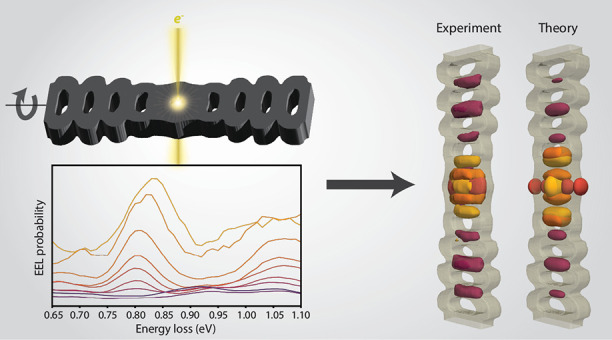

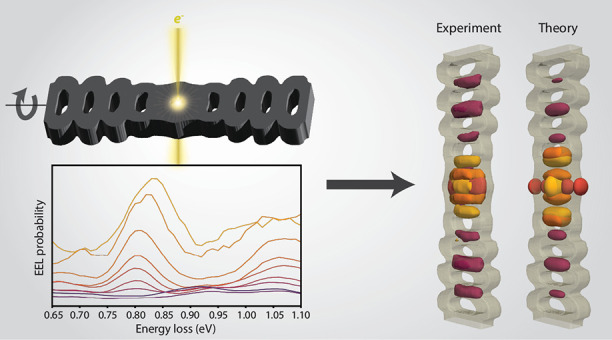

The light–matter interaction forms the foundation for a plethora of research fields, including photonics, materials science, and sensing.^1–3^ The ability to control and enhance this interaction is considered to be crucial for technological advancements in applications ranging from low-power and ultrafast optoelectronics^4,5^ to ultrasensitive chemical detection^6^ and emerging quantum technologies.^7^ The interaction can be enhanced within a resonant optical cavity, characterized by its mode volume (*V*_eff_), which represents the spatial confinement of light, and its quality factor (*Q*), which describes its ability to store light. As a prominent example, the rate of spontaneous emission of a point-like emitter inside the cavity is quantified by the Purcell factor, $F_{P}=\frac{3}{4\pi^{2}}{\left(\frac{\lambda }{n}\right)}^{3}\left(\frac{Q}{V_{\mathrm{eff}}}\right)$ , where λ is the wavelength and *n* is the refractive index.^8^ Plasmonic nanomaterials, such as gold and silver, are promising candidates for spatially confining light to sub-wavelength volumes through coupling to their free electron gas.^9–11^ The same effect, however, leads to significant losses from dissipative damping processes, which limits the achievable quality factors to below 100.^12^ Dielectric materials, in contrast, can provide photonic cavities with much larger quality factors, but their mode volume has traditionally been on the order of the cube of the wavelength, which has previously led to speculations on whether there might even be an Abbe–Rayleigh-type limit to their ability of spatially confining light.^13^ Through numerous counter examples it is by now well-documented that there is no such limit, and there have recently been substantial efforts to promote the development of dielectric photonic nanostructures for confining light to deep sub-wavelength dimensions.^14–20^ A promising approach used in this study is the inverse design by topology optimization,^21,22^ which can generate structures with extreme spatial confinement of light while maintaining relatively high quality factors.^18,23,24^

As mode volumes shrink, the demand for reliable nanometer-scale imaging and analysis of optical fields grows in parallel. Scattering scanning near-field optical microscopy (s-SNOM) has previously been used to characterize these structures but is approaching its limits in terms of spatial resolution,^17,18,24^ and its probing tip can introduce perturbations to the fields under examination, leading potentially to misinterpretation of the results.^25^ Moreover, s-SNOM is performed on planar surfaces and thus cannot provide volumetric information about mode confinement. Recently, nanometer-resolution 3D tomographic and vectorial near-field imaging deep inside dielectric materials has been demonstrated using high-order sideband generation.^26^ Complementary to these optical techniques, an alternative route to probing optical excitations with even higher spatial resolution is offered by free electrons in scanning transmission electron microscopy (STEM).^27^ In particular, the application of electron energy-loss spectroscopy (EELS) in STEM to visualize the plasmonic modes of metallic nanostructures under various conditions, including their three-dimensional (3D) imaging, has been widely reported.^28–33^ In comparison, only a few examples of EELS imaging of optical modes in dielectric photonic cavities can be found in the literature.^34–37^ To date, these investigations have primarily involved planar photonic crystal cavities consisting of arrays of cylindrical holes. The geometry of these structures, however, does not allow the acquisition of a full tilt series to conduct tomographic reconstruction of the optical modes. The closest example of such a study is a recent elegant use of a high-*Q* photonic crystal cavity slab with optical modes probed by the electron beam from two perpendicular directions.^37^ Despite these advances, successful 3D imaging of optical modes and, thereby, verification of sub-wavelength field confinement in dielectric photonic cavities has so far proven challenging. Here, we report on the EELS tomographic reconstruction of the optical modes of a 220 nm thick silicon nanobeam bowtie cavity optimized to support a resonant mode with sub-wavelength confinement inside the material at its center.^38,39^ Due to the geometry of the nanobeam, we were able to access and image the bowtie cavity over a wide range of rotation angles around its longitudinal axis, thereby enabling 3D characterization of polarized modes. Our results uncover the modal structure of the nanocavity, including a resonant mode at ∼1550 nm confined to the center of the cavity, in excellent agreement with the intended design and simulations.

Here, we adopt a comprehensive approach that includes design, fabrication, characterization, and theory. In our study, a waveguide-coupled bowtie optical cavity supporting a resonant mode of interest (MOI) at 1550 nm with *Q* = 600 and *V*_eff_ = $0.32{\left(\frac{\lambda }{2n}\right)}^{3}$ was designed using topology optimization,^39^ corresponding to a Purcell factor of 1.1 × 10^3^. Subsequently, arrays of nominally identical clones of the structures were fabricated by using electron beam lithography and dry etching. Details of the design and fabrication process are described in sections S1 and S2 of the Supporting Information.

A top-view scanning electron microscopy (SEM) image of a representative structure is shown in Figure 1a. Each structure consists of a bowtie nanocavity, flanked by a series of holes on either side, embedded in a waveguide, and terminated at each end by grating couplers. The grating couplers facilitate optical transmission measurements, and based on the spectra shown in Figure S1, the clones of the structure studied in this work have an average resonance wavelength of 1520.2 ± 5.1 nm (0.82 ± 0.1 eV) and quality factor of 870.5 ± 188.0. To examine the samples in transmission electron microscopy (TEM), individual nanocavity beams were cut out and transferred to a TEM-compatible grid using a plasma-focused ion beam (PFIB) instrument. A SEM image of the cut-out nanobeam cavity is presented in Figure 1b, and details of the sample transfer process are presented in section S3 of the Supporting Information. As shown in Figure S2, the structures were attached to a TEM grid in two complementary orientations: one with the bowtie in edge-on and the other in plan-view orientation. This made it possible to access the structure over a full 90° tilt range around its longitudinal axis in TEM. Key dimensions of the bowtie structure, including the thickness of the native oxide layer and size of the bowtie bridge, were determined using energy-dispersive X-ray spectroscopy (EDS) and high angle annular dark field (HAADF) STEM imaging, revealing a crystalline bowtie bridge size of approximately 7 nm (see Figure 1c and Figure S3 for details). The nanocavity was designed with the MOI closely confined to the bowtie region and polarized along the direction of the silicon bridge, defined as the *x* direction. Independent numerical finite-element calculations were carried out based on the experimentally measured dimensions of the structure (Figure 1c and d and Figure S3). They confirm this confinement and provide the full 3D vectorial information on the MOI as well as additional modes supported by the fabricated structure. Isosurface renderings of the *x*-polarized normalized electric field strength, |**E**_*x*_|, of the MOI are displayed in Figure 1e.

**Figure 1:**
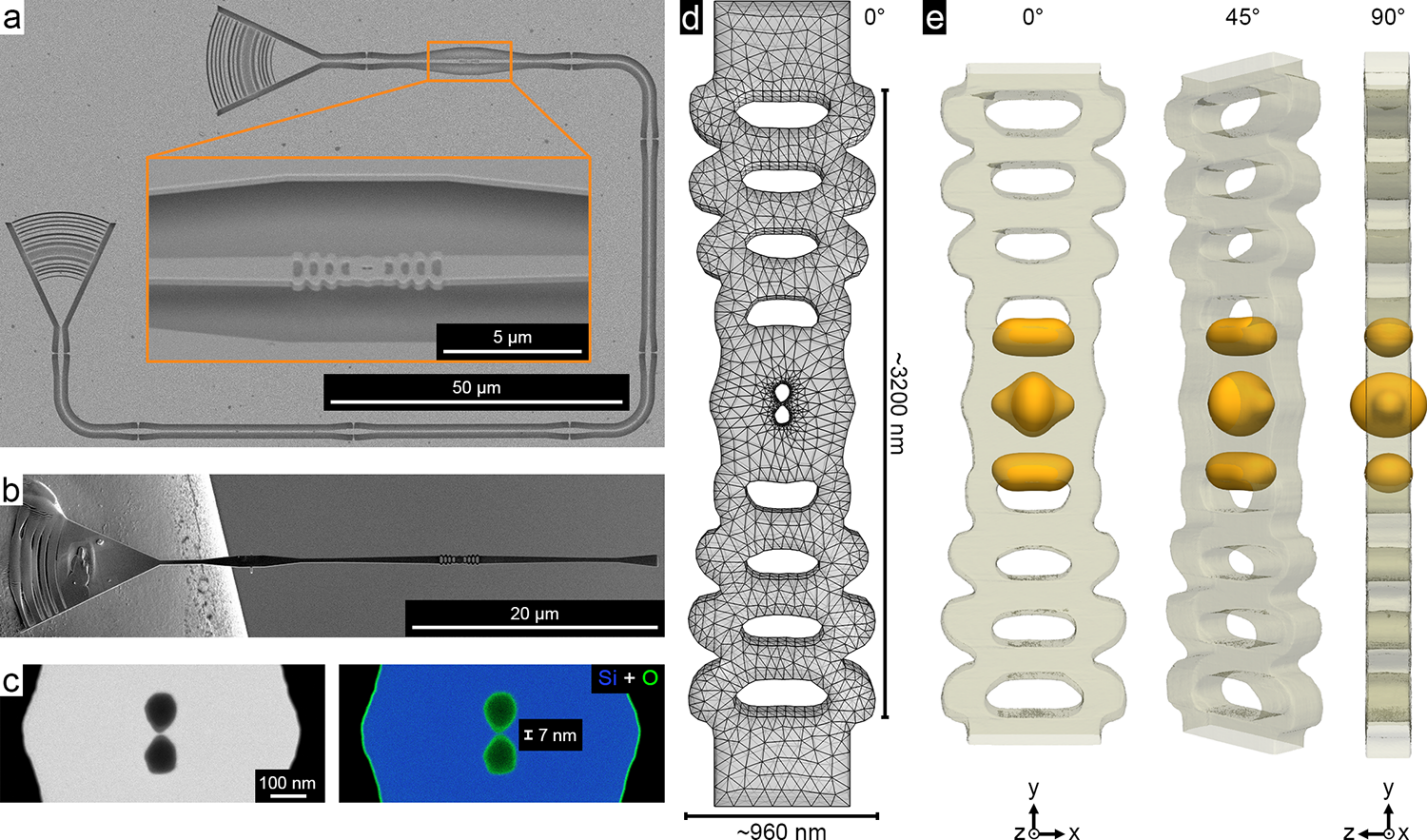
Overview images of the waveguide-coupled TO bowtie cavity designed to have a resonant mode at 1550 nm, confined to the bowtie region and polarized along the *x* direction. (a) Top-view SEM image generated by backscattered electrons revealing the fabricated dielectric photonic cavity attached to grating couplers. The inset shows a side view of the bowtie cavity after tilting the sample to 30°. (b) Fabricated structures were lifted out of the substrate and attached to a TEM grid. (c) STEM image of the bowtie region of the cavity and EDS chemical map of silicon and oxygen. (d) Mesh for the numerical model of the TO structure with a thickness of 220 nm and further relevant sample dimensions. (e) Isosurface renderings of normalized |**E**_*x*_| calculated for the MOI and plotted using a 35% threshold. A full description of the calculations can be found in section S5 of the Supporting Information.

To probe the optical modes of the nanocavity experimentally, we recorded EEL spectra from the bowtie region at different rotational angles θ, as illustrated in Figure 2a. The processed experimental spectra are listed in Figure 2b. The swift electrons in EELS interact with the electric field component of an optical mode aligned along their trajectory.^35,40–42^ Thus, the interaction between the electrons and the *x*-polarized MOI and, consequently, the intensity of the EELS signal at its resonance energy are expected to increase when rotating the sample toward the edge-on geometry. This effect is evident in the EEL probability density spectra: the peak at ∼0.82 eV is absent at 0° tilt but grows progressively in intensity with increasing tilt. Although a maximum EEL signal is expected at 90° tilt, the spectrum from this orientation is not included due to a poor signal-to-noise ratio resulting from electrons traveling through approximately 700 nm of material and the sample’s alignment along a crystallographic zone axis, which likely increased elastic scattering. Many factors, therefore, contribute to defining the practical geometrical limits of dielectric structures accessible to the imaging approach presented here.

**Figure 2:**
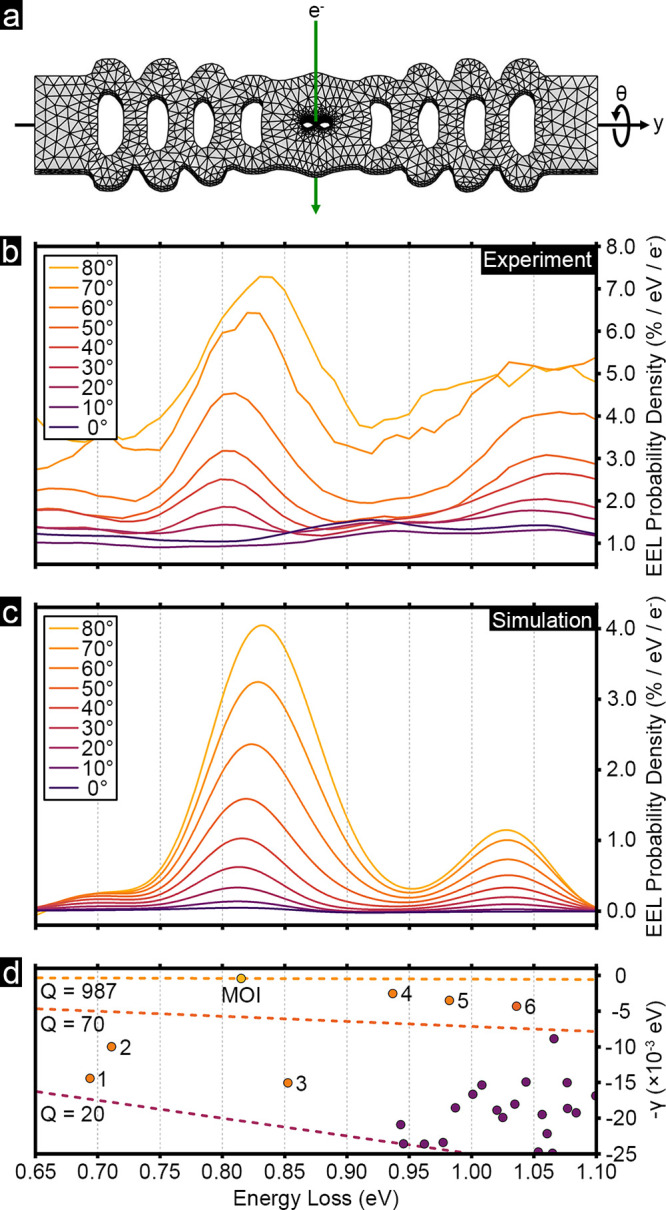
EELS signal from the center of the bowtie at different rotation angles. (a) Schematic showing the configuration of the electron beam relative to the nanobeam cavity at different rotation angles, θ. (b) Experimentally obtained EEL probability density spectra with the electron beam passing through the center of the bowtie cavity at different angles, where θ = 0° corresponds to the plan-view geometry. (c) Simulated EEL probability density spectra from the center of the bowtie cavity at different tilt angles. (d) Spectrum showing the discrete distribution of the simulated QNM energies in the complex plane, highlighting the most prominent modes of the cavity, including the MOI and modes 1–6. The dashed lines denote the threshold for *Q* > 1000, *Q* > 70, and *Q* > 20, respectively. These thresholds were used to identify modes 1–6 as prominent contributing factors to the EELS probability density spectra in panels b and c.

We used a modal description to analyze and explain the observed resonances in the EEL spectrum (details can be found in section S5 of the SI). Moreover, we calculated the quasinormal modes (QNMs),^43–46^ also known as resonant states,^47,48^ and simulated the EEL spectra following the description given by Ge and Hughes.^41^ The QNMs have complex frequencies of the form ω̃ = ω_*n*_ – *i*γ_*n*_, with γ_*n*_ > 0, in which the imaginary part accounts for dissipation of energy, giving the quality factor of each resonance as *Q*_*n*_ = ω_*n*_/2γ_*n*_. The simulated EEL spectra are shown in Figure 2c along with the calculated spectrum of QNM resonance frequencies in the complex plane in Figure 2d. The QNM corresponding to the MOI has a resonance energy of 0.815 eV and a quality factor of 1009, which agrees well with the optical measurements (cf. section S2 of the Supporting Information). The corresponding effective mode volume is *V*_eff_ = $0.19{\left(\frac{\lambda }{2n}\right)}^{3}$ , which is lower than that of the design primarily due to the presence of SiO_2_ inside and around the bowtie region. A closer look at Figure 2d suggests that, while the MOI has by far the highest quality factor, other lower *Q* modes also exist. We identified six additional modes that contribute substantially to the optical response of the cavity, marked with numbers 1–6 in Figure 2d: those with resonance energies in the range of 0.65–0.90 eV and *Q* ≥ 20 and those with resonance energies within the range of 0.90–1.10 eV and *Q* ≥ 70. Simulating EEL probability density spectra from the bowtie region based on the seven identified QNMs successfully reconstructs the main features of the experimental data, as seen in Figure 2c. Interestingly, the slight shift in the spectral position of the main peak with the tilt angle is reproduced by the theoretical model and can be understood to be due to contribution from neighboring mode 3. A comparison between Figure 2b and c also reveals a tilt-dependent background in the experimental data, which we attribute to the effect of low-*Q* modes not included in the simulations as well as other possible energy-loss mechanisms present in the experiment.

Next, we spatially mapped these EELS spectral features by acquiring spectrum images incorporating two spatial axes and one energy-loss axis. To extract the spectral signatures of different components in the EELS data and their spatial distributions, we applied non-negative matrix factorization (NMF),^29,49^ with the details of the data processing described in section S6 of the Supporting Information. This procedure successfully extracts the seven modes identified by the QNM analysis as the main components present in our experimental data (see sections S6 and S7 of the Supporting Information for details). Although the signal-to-noise ratio becomes too poor at 90° tilt to yield meaningful EEL spectra from the bowtie cavity, this orientation allows probing the evanescent field of the modes in vacuum. Figure 3 shows normalized EEL probability maps for different modes alongside a HAADF STEM image of the fabricated structure. In these maps, the nanobeam region is masked and split-view comparisons of experimental versus simulated normalized EEL probability maps are presented. In most cases, we find a good correspondence between experimental and simulated EEL probability maps. In particular, the experimental profile of the MOI in Figure 3a closely matches both the simulated data and normalized |**E**_*x*_| in Figure 1e. The two highest *Q* modes, namely, MOI and mode 4, exhibit distinct spatial distributions, with the MOI confined at the bowtie center and mode 4 flanking the bowtie (see Figure 3b). This is clearly reflected in the EEL probability density spectra shown in Figure 3c acquired from different positions along the nanobeam cavity, and the simulated spectra accurately reproduce this trend. Nonetheless, the maps do not provide clear distinctions between all of the modes, with, e.g., the MOI and mode 3 looking very similar in Figure 3a. This highlights the limitations of single-angle imaging and underscores the necessity of acquiring data at multiple rotation angles to obtain 3D information about the modes.

**Figure 3:**
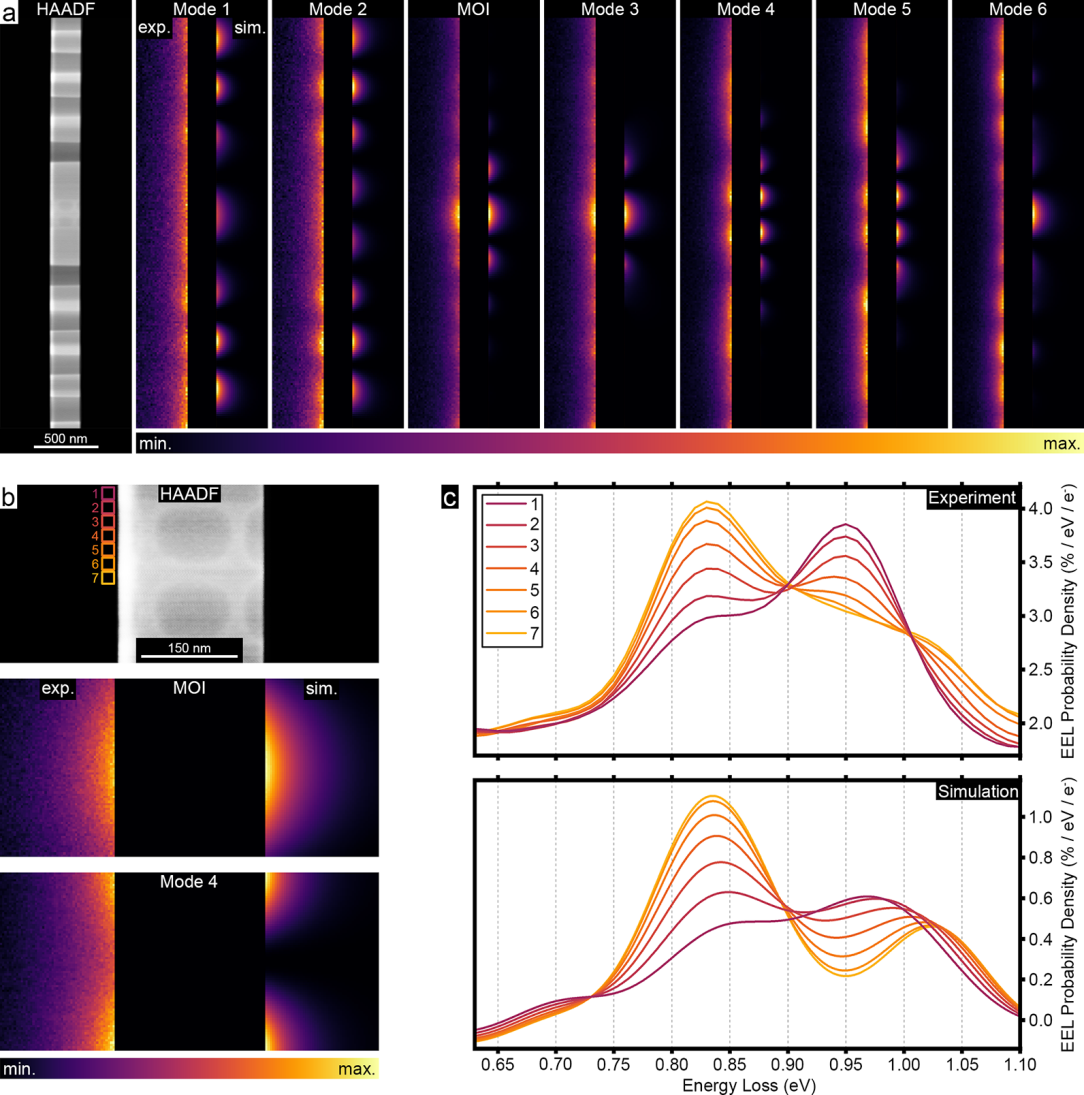
EEL probability maps of the MOI and modes 1–6 at 90° specimen tilt. (a) HAADF–STEM image of the structure with a 90° tilt and normalized EEL probability maps of the MOI and modes 1–6 extracted from the NMF analysis of the experimental (left-hand side) and simulated (right-hand side) data. The silicon region in the image has been masked to focus on the evanescent fields of the modes in vacuum. (b) EEL probability maps of the MOI and mode 4 at higher magnifications alongside their corresponding simulated maps, highlighting their distinct spatial profiles. Note that, for each individual map, the minimum value corresponds to the lowest EEL probability and the maximum value corresponds to the highest EEL probability within that map. The specific limits for each intensity bar are provided in Table S1 of the Supporting Information. (c) EEL probability density spectra extracted from seven different positions close to the bowtie center as marked in the HAADF image in panel b along with simulated spectra for the same regions.

To study the spatial distribution of the optical modes in the plane of the sample, we analyzed EEL probability maps of the sample acquired over the 0–90° tilt range. Signals from the modes in maps taken at 0–30° and 90° were too weak or noisy for accurate analysis and were therefore excluded. Figure 4a–c presents the experimentally measured and simulated normalized EEL probability maps of the MOI at 40–80° tilts, alongside STEM images. Corresponding maps for modes 2–4 can be found in section S7 of the Supporting Information. In general, we find a convincing agreement between the experiment and theory, further demonstrated in the higher resolution EEL probability map of the bowtie region at a 60° tilt in Figure 4e. Notably, the EEL probability line profiles plotted along the nanobeam’s long axis (*y* direction) in Figure 4d show excellent agreement between experimental and simulated data, indicating that the MOI’s effective mode volume, *V*_eff_, closely matches its theoretical prediction.

**Figure 4:**
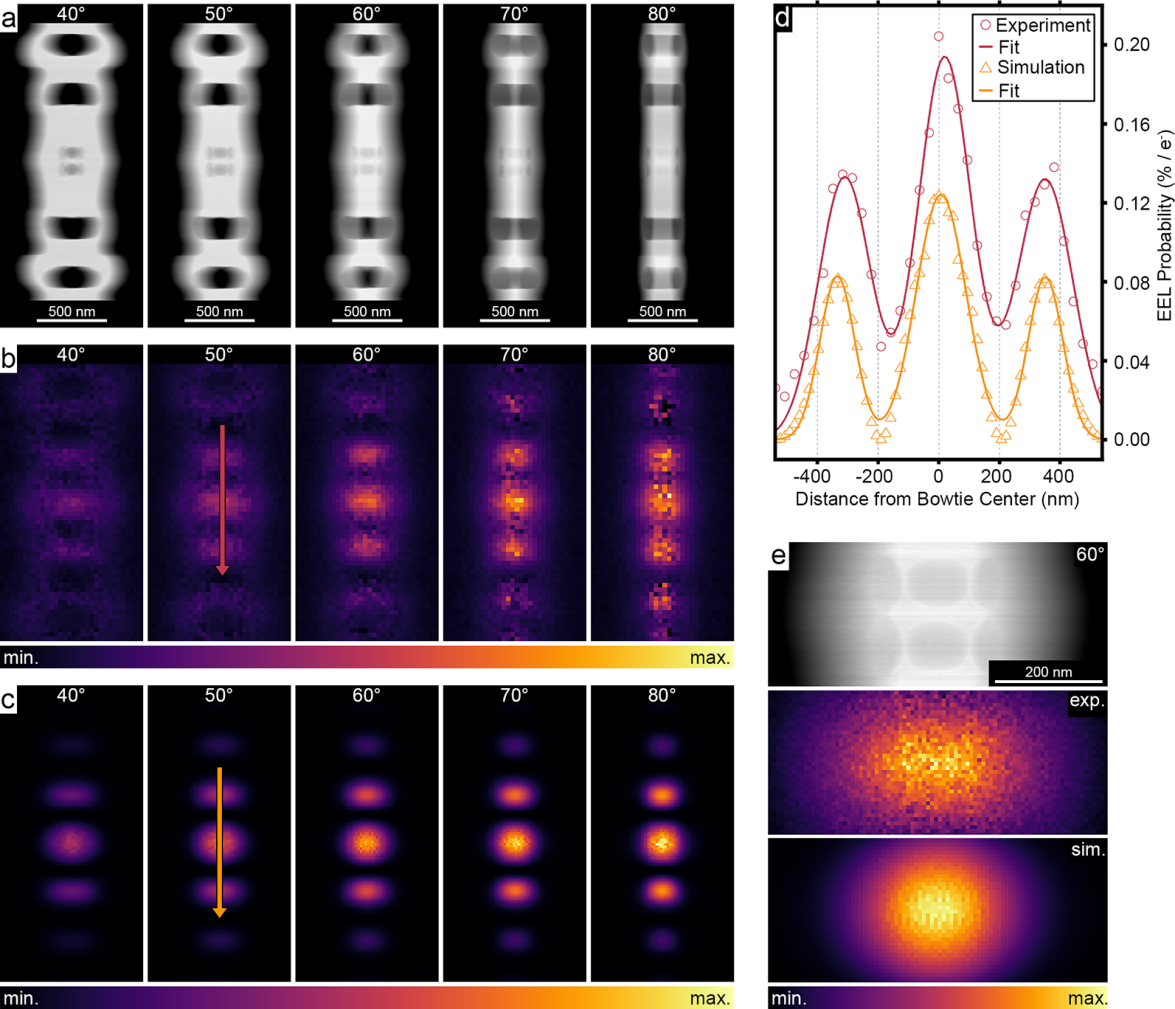
EEL probability maps of the MOI at different specimen rotation angles. (a) HAADF–STEM images of the nanobeam cavity at different tilt angles in the range of 40–80°. (b) Experimental and (c) simulated normalized EEL probability maps of the MOI at 0.82 eV at each corresponding orientation. Note that, for each tilt series, the minimum value corresponds to the lowest EEL probability and the maximum value corresponds to the highest EEL probability across all maps in the series. (d) Line profiles of the normalized EEL probability maps along the arrows shown in panels b and c for the structure at 50° tilt. (e) High-magnification experimental and simulated normalized EEL probability maps of the MOI at 60° tilt. Note that, for each individual map, the minimum value corresponds to the lowest EEL probability and the maximum value corresponds to the highest EEL probability within that map. The specific limits for each intensity bar are provided in Table S1 of the Supporting Information.

Our EELS analysis of the freestanding nanobeam structure enables probing of the cavity’s *x*-polarized optical modes along arbitrary rotation angles around its longitudinal axis, thereby raising the prospect of recovering their 3D profiles. Conventional tomographic reconstructions are based on performing an inverse radon transform of 2D projections of a 3D object acquired over multiple viewing angles. In our case, the 3D entity to be reconstructed refers to the electric field vector associated with the optical modes of the cavity. Although theoretical frameworks for vector-field electron tomography have been established and typically require tilting the structure around more than one axis,^50,51^ their application to time-varying fields demands correspondingly high temporal resolution. Alternatively, the EELS signal in conventional TEM can be interpreted in terms of the spatial envelope of the resonant modes.^35^ It has been argued that, under certain conditions, EELS can be described as Δ*u*(**R**_θ_, ω) ∼ cos^2^(90° – θ)| R _θ_(**f̃**_*n*_(**r**))|^2^, where Δ*u*(**R**_θ_, ω) is the spectral energy-loss along the electron path, defined by the position in the image plane **R**_θ_ at the sample rotation angle θ, R _θ_ denotes radon transformation, and **f̃**_*n*_(**r**) refers to the amplitude of the QNMs of the structure.^28,29,35^ As detailed in sections S5 and S8 of the Supporting Information, this simple expression is valid in the limit of a very small spatial extent of the optical response and for purely real modes. Under these conditions, tomographic reconstruction of the tilt series can recover the 3D profiles of the considered polarized optical modes. Although these criteria are not strictly met in our experiment, we find that the reconstructed fields agree remarkably well with the calculated mode profiles.

Given the *x*-polarized nature of the MOI by design, our single tilt series of EELS maps provides the projection data required for a tomographic analysis. Accordingly, the normalized EEL probability maps were used to reconstruct 3D images of the MOI as well as modes 2–4, as displayed in Figure 5a together with 3D reconstruction of the cavity structure based on the HAADF STEM images. These tomographic reconstructions should be compared to the normalized |**E**_*x*_| of the corresponding calculated modes, as shown in Figure 5b. Overall, the EELS tomography reconstructions are in excellent agreement with the theoretical profiles of normalized |**E**_*x*_| of the investigated modes, with many key features successfully captured in the reconstructions. Some of the finer features of the resonant modes have not been generated in the reconstructions; specifically, the profile of the MOI is artificially elongated along the *x* direction and has a smaller extension into the surrounding vacuum. In tomography, a lack of access to projected images along a range of tilt angles, known as the missing wedge,^52^ is expected to lead to artificial elongation of the reconstruction in the direction of the missing wedge. In our case, the missing wedge includes tilt angles below 40°, and is likely giving rise to the elongation observed here. Additionally, inaccuracies in background signal removal, described in Figure S15, likely also contribute to the underestimated signal in a vacuum. Other small discrepancies could have risen due to difficulties in isolating the contribution of spectrally overlapping modes (e.g., MOI and mode 3).

**Figure 5:**
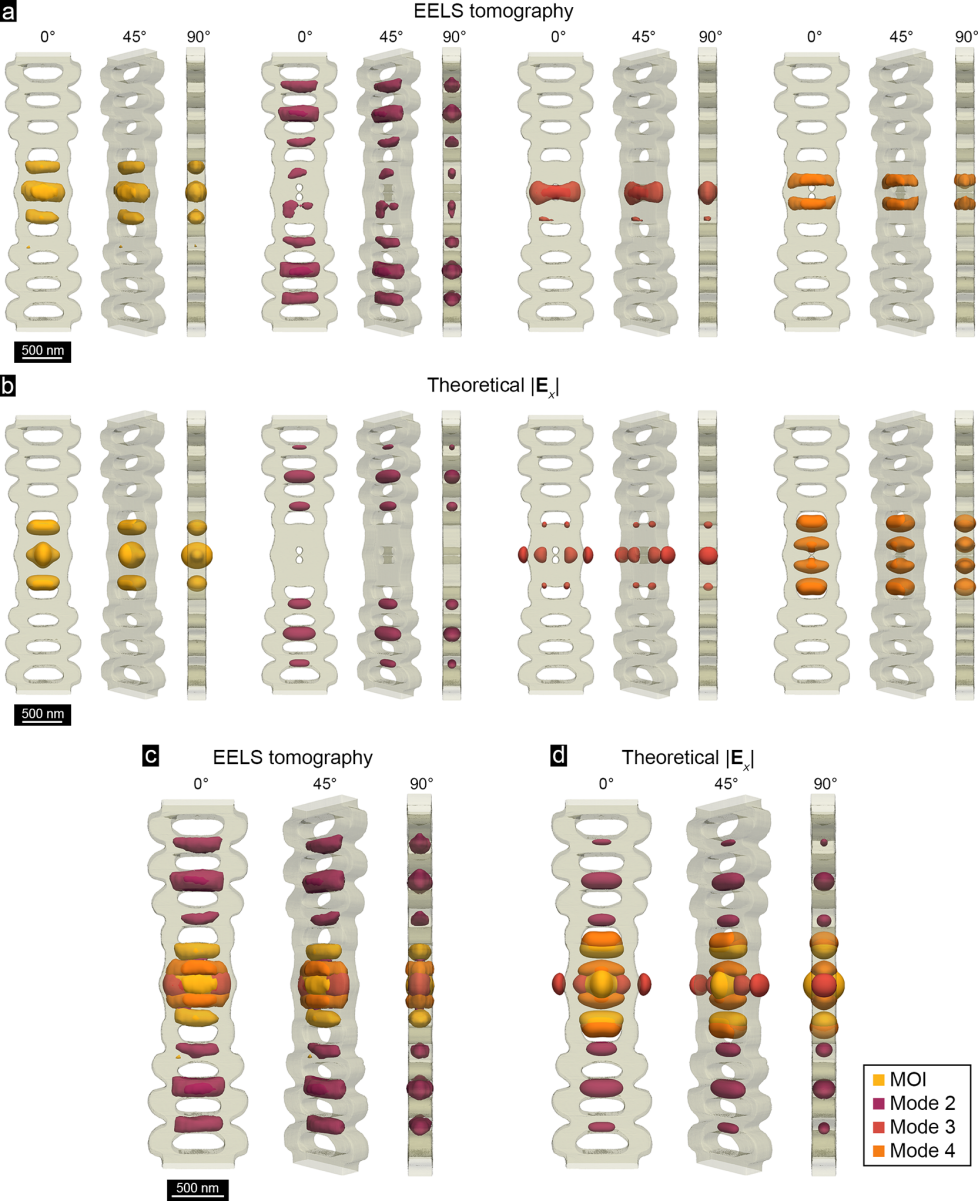
3D visualization of the MOI and modes 2–4. (a) Isosurface renderings of the 3D profiles of the MOI and modes 2–4 at 35% of the MOI’s |**E**_*x*_| maximum, obtained from tomographic reconstructions of the experimental EEL probability maps. Profiles for each mode are shown at 0°, 45°, and 90° rotations about the long axis of the nanobeam cavity. (b) Isosurface renderings of theoretically calculated |**E**_*x*_| for the MOI and modes 2–4 at 35% of the MOI’s |**E**_*x*_| maximum. (c and d) Overlayed 3D profiles of the MOI and modes 2–4 obtained from EELS tomography and theoretical calculations.

Overall, our results confirm the theoretical predictions that EELS probes the **E**-field component of optical fields along the direction of the electron beam and, when combined with tomography, can recover the full 3D profile of resonant optical modes in nanostructures under specific experimental conditions that permit adequate angular coverage. Pertinent to this investigation, the close agreement between the reconstructed and theoretical 3D profiles of the MOI corroborates our calculations of a sub-wavelength mode volume. To our knowledge, this study presents the first 3D reconstruction of sub-wavelength optical fields in dielectric cavities using a free electron-based technique. The ability to concentrate light into sub-wavelength volumes promises transformative advances in integrated photonic circuitry, from ultrafast signal processing to on-chip quantum optics. Here, we demonstrate that EELS-based tomographic reconstruction yields volumetric field profiles in close agreement with the theoretical profile of the modes. In combination with standard optical characterization, which can be done on the exact same nanostructure by using our focused ion beam lift out and transfer process, this provides a powerful high-resolution imaging tool for current and future nanophotonic devices. Moreover, our method is readily compatible with complementary electron beam spectroscopic methods, such as electron energy-gain spectroscopy (EEGS) and cathodoluminescence (CL), adding fascinating new layers of control for imaging cavity modes with deep sub-wavelength field confinement. Indeed, the waveguide-coupled nature of the cavity under study can, in principle, be used to pump the cavity with an external laser source for EEGS or to collect the light generated in the cavity by the free electrons for CL. Since both effects are governed by physical processes, like those in EELS, the same framework is applicable with small modifications, and by coupling through the waveguides, one can conveniently change between the three techniques *in situ* or even perform simultaneous measurements.

## Supplementary Material







## Data Availability

Data used for the article are currently available at https://doi.org/10.11583/DTU.29672895.

## Notes

Details of the design, nanofabrication and optical characterization, FIB sample transfer, TEM analysis, theoretical simulations, EELS data processing, comparison of experimental and simulated EELS maps, and tomography (PDF)
Isosurface rendering of theoretically calculated |**E**_*x*_| (GIF)
Isosurface rendering of EELS tomography reconstructions (GIF)
