# Supplementary material for: 3D Imaging of Optical Modes in Dielectric Photonic Nanocavities with Sub-wavelength Field Confinement
Source: Nano Lett. 2025 Nov 10;25(46):16395–403. doi: 10.1021/acs.nanolett.5c04226 (PMC12636085; doi:10.1021/acs.nanolett.5c04226)
Supplement: Supplementary file 1 [file nl5c04226_si_001.pdf]

Supporting Information

for

3D Imaging of Optical Modes in Dielectric Photonic Nanocavities with Sub-wavelength Field Confinement

Michael S. Seifner[1,4], Anne Sofie Darket[2,4], Ali N. Babar[2,4], Babak Vosoughi Lahijani[2,4], Rasmus E. Christiansen[3,4], Ole Sigmund[3,4], Elizaveta Semenova[2,4], Søren Stobbe[2,4], Philip T. Kristensen[2,4], Shima Kadkhodazadeh[1,4]*

[1] DTU Nanolab, Technical University of Denmark, Fysikvej 307, 2800, Kongens Lyngby, Denmark

[2] DTU Electro, Technical University of Denmark, Ørsteds Plads 343, 2800, Kongens Lyngby, Denmark

[3] DTU Construct, Technical University of Denmark, Koppels Allé 404, 2800, Kongens Lyngby, Denmark

[4] NanoPhoton – Center for Nanophotonics, Technical University of Denmark, Ørsteds Plads 345A, 2800, Kongens Lyngby, Denmark

* shka@dtu.dk

<u>Address:</u>

Fysikvej, 307, 118

2800 Kongens Lyngby

Denmark

<u>E-mail:</u>

shka@dtu.dk

S1. Design

The photonic cavity studied in this work was designed according to density-based topology optimization using a finite element model of electromagnetics assuming time-harmonic field behavior.[1] The inverse design framework includes two geometric length-scale constraints, that enables specification of the minimum size of all features in each material phase that must be respected in the design process for the device blueprint.[2] The length-scale can be tuned to fit a particular fabrication method, ensuring that no blueprint will be designed that cannot be fabricated. Further, the framework includes a connectivity constraint[1,3] that ensures that all solid features in the design are connected to a pre-specified region of the design domain, ensuring that all device blueprints are mechanically self-supporting and thus manufacturable as membranized devices. The objective was to maximize the electric field magnitude at the center of a 220 nm thick silicon waveguide for $\lambda = 1550$ nm (0.80 eV), ensuring a spatially localized, resonant optical mode. The constraints applied in the design ensured a final device spatially connected to input and output waveguides, exhibiting a minimum feature size amenable to fabrication by electron beam lithography, and supporting high transmission of light through the waveguide at the operating wavelength.[4]

We note that, while for this study we targeted the above design objectives, the inverse design framework allows for targeting arbitrary functionals of the electromagnetic field. As a consequence, one could employ the design framework to tailor resonators to accommodate a wide range of both electric and magnetic modes by changing the figure of merit accordingly and carrying out the appropriate adjoint sensitivity analysis[5] to compute the sensitivities (gradients) for use in solving the optimization problem.

S2. Nanofabrication and optical characterization

Fabrication

The nanocavity device was fabricated on a double-sided polished silicon-on-insulator (SOI) substrate, with a silicon device layer thickness of 220 nm. A double hard mask was used to etch the nanocavity with high fidelity, transferring the pattern from the resist into the 220 nm thin silicon device layer. To this end, a layer of 30 nm chromium followed by 12 nm poly-silicon was deposited on the silicon device layer using sputtering. An electron beam lithography process was used to transfer the nanocavity design pattern directly into a 50 nm-thick electron-sensitive resist, deposited using spin coating, on a substrate coated with a hard mask. The details of the hard mask etching process can be found elsewhere.[4] Subsequently, the pattern was etched into the silicon device layer using deep reactive-ion etching, which utilizes SF_6 and O_2 as etch and passivation gases. The process is capable of selectively and directionally etching the pattern with high accuracy and fidelity. The etching of the silicon device layer was followed by removal of the hard mask stack inside the reactive ion etching tool. After transferring the pattern into the substrate, the 220 nm thin Si membrane was suspended by removing the 2 μm buried thermal oxide (BOX) layer using anhydrous vapor-phase hydrofluoric acid etching. Details of the fabrication process can be found elsewhere.[4]

Optical transmission measurements

The spectral properties of the fabricated devices were characterized using a far-field optical microscope. The orthogonal arrangement of the grating couplers attached to the waveguides enabled spatially resolved and cross-polarized confocal optical measurements. To determine the mean resonance frequencies of the fabricated devices with identical nominal dimensions, the end-to-end absolute power transmissions from the nanocavities were measured in the wavelength range 1480 nm-1640 nm by coupling a monochromatic, tunable external cavity diode laser (Santec TSL-710) into the photonic circuit via one of the grating couplers. A detailed description of the optical setup and characterization can be found elsewhere.[6] The results from seven clones of the investigtated topology-optimized (TO) bowtie cavity are presented in Figure S1, revealing the spectral signature of the mode of interest (MOI) at approximately 1550 nm. A statistical evaluation of the measured peaks gives an average resonance wavelength of 1520.2 nm ± 5.1 nm for this mode and a quality factor (Q) of 870.5 ± 188.0.

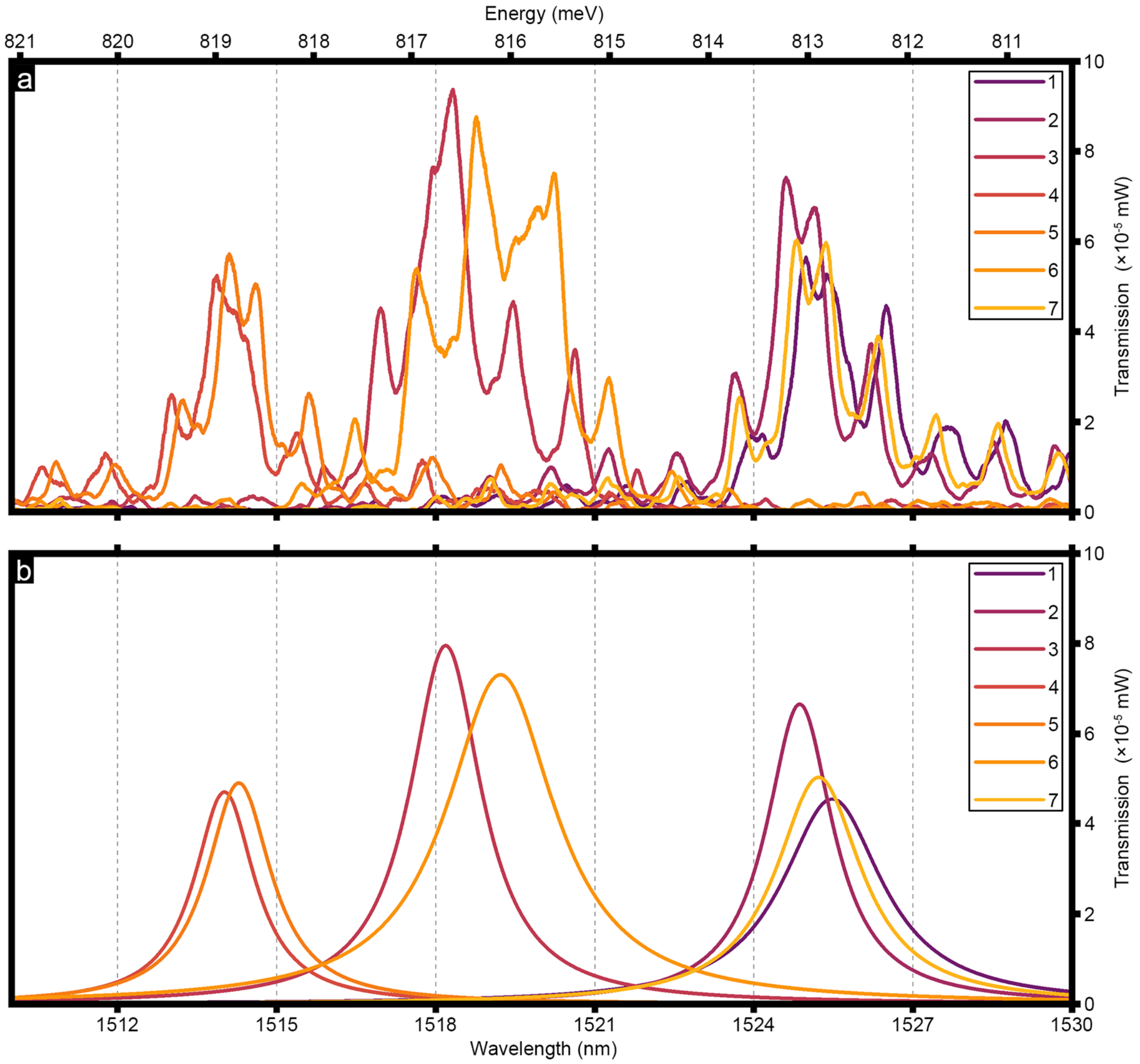

Figure S1: (a) Transmission measurements of seven clones of the TO bowtie cavity structure. (b) Each measurement in (a) was fitted with a Lorentzian peak. Based on the fits, the statistical evaluation yielded a resonance wavelength of 1520.2 nm ± 5.1 nm and a quality factor of 870.5 ± 188.0.

S3. FIB Sample transfer

Samples for investigation in the transmission electron microscope (TEM) were prepared using a dual-beam plasma focused ion beam/scanning electron microscope (PFIB/SEM) instrument (Thermo Fisher's Helios 5 Hydra Ux DualBeam). SEM images were acquired with an accelerating voltage of 2 kV-5 kV and an electron current of 100 pA-200 pA using secondary or backscattered electrons as signals. A xenon ion beam (accelerating voltage: 30 kV, ion current: 10 pA) was used to cut the thin attachment tethers around the sample and release the nanobeam cavity. The sample was then lifted out from the substrate by welding a tungsten needle to the edge of the sample and attached to a TEM-compatible grid. Two clones of the TO bowtie cavity structure were attached to the same TEM grid with different orientations to cover the tilt range of 0°-90°. The plan-view orientation was used to access the 0°-20° tilt range, while the edge-on orientation was viewed in the 30°-90° tilt range. Details of this transfer process are shown in Figure S2.

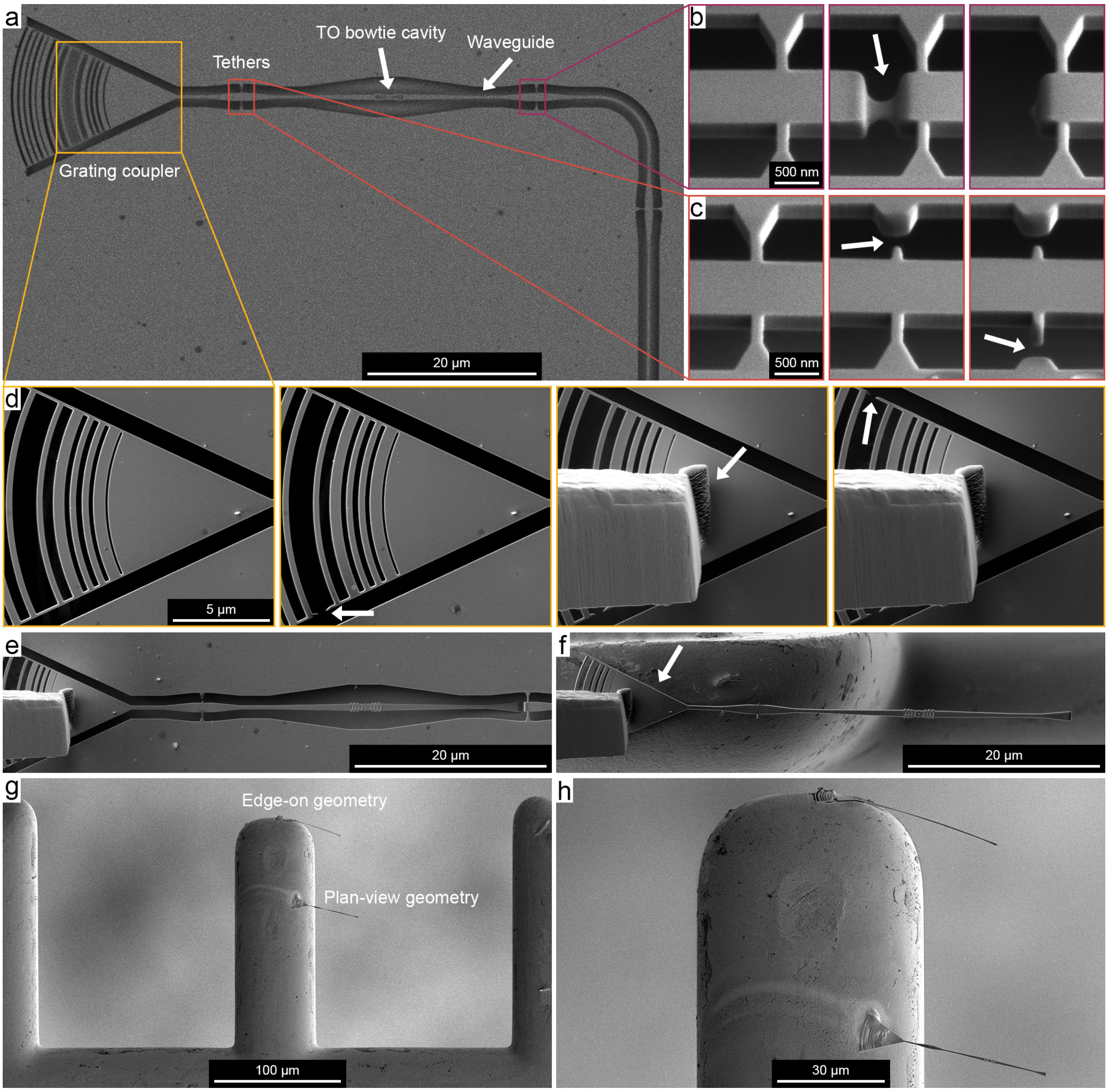

Figure S2: (a) Overview SEM image of a silicon TO bowtie cavity embedded in a waveguide and attached to grating couplers. (b-d) During the PFIB sample transfer, the waveguide (dark magenta rectangle in (a)), the tethers (red rectangle in (a)), and part of the grating coupler (orange rectangle in (a)) were cut by a xenon ion beam. (d) The structure was welded to a needle micromanipulator and the grating coupler was then cut to release the structure. (e) The attached structure was lifted out, (f) moved, brought in contact with a TEM grid, and welded to it (indicated by arrow). The needle was then cut free from the sample by the xenon ion beam and retracted. (g-h) The process was applied to attach the sample to the TEM grid in both edge-on and plan-view geometries.

S4. TEM analysis

An aberration-corrected Ultra Spectra (S)TEM (Thermo Fisher) was used for the study. The microscope is equipped with a monochromator, an Ultra-X energy dispersive X-ray spectroscopy (EDS) system and a Continuum HR/1066 30-300 kV Gatan GIF system for electron energy-loss spectroscopy (EELS) data collection. The measurements were recorded in scanning transmission electron microscopy (STEM) mode and with the monochromator excited, set up to give an electron probe with a convergence semi-angle of 18.5 mrad, a probe current of approximately 150 pA, a probe diameter of approximately 120 pm, and an energy resolution better than 100 meV. The EEL spectrum images were acquired with a spectrometer collection semi-angle of approximately 8 mrad. Two different sets of EEL spectrum images were acquired for each sample tilt. The first set of measurements focused on the whole structure and used a pixel size of approximately 2.0 nm × 2.0 nm and an acquisition time of 1 ms per pixel. The second set of measurements focused on the bowtie region and used a pixel size of approximately 0.5 nm × 0.5 nm and an acquisition time of 1 ms per pixel. The sample was tilted in 10° increments, and at each step, high-angle annular dark-field (HAADF)-STEM images and EEL spectrum images were recorded.

Details of the chemical and structural characterization of the TO cavities are shown in Figure S3. HAADF-STEM imaging and EDS were used to obtain measurements of the key features of the structures studied in the main manuscript, including the thickness of the native oxide layer and the length of the crystalline silicon in the central bridge of the bowtie cavity.

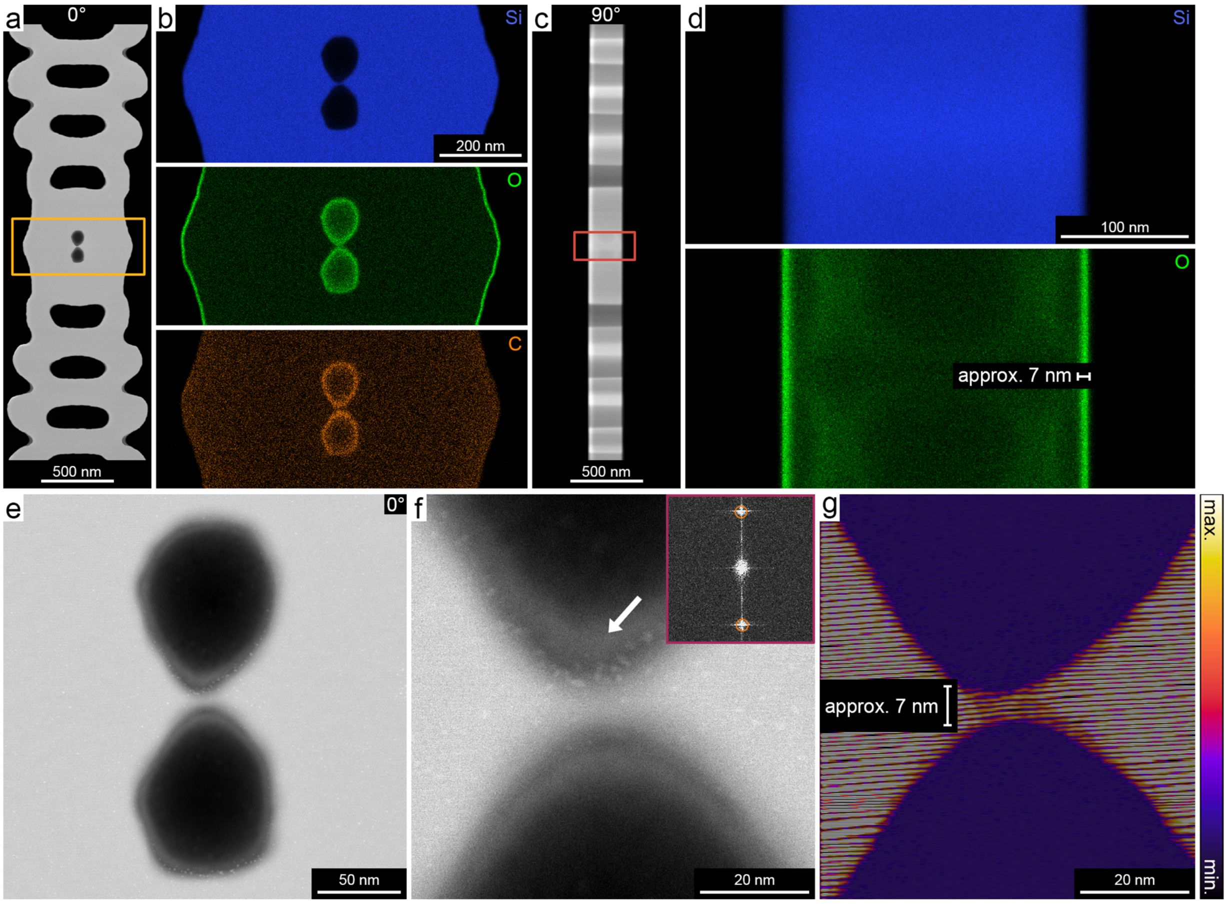

Figure S3: (a) HAADF-STEM image of the TO bowtie cavity at 0° tilt. (b) EDS intensity maps of Si, O, and C from the region marked in (a), recorded after the EELS data acquisition. The data confirms the presence of oxide surfaces and some C contamination on the surfaces and in the holes of the bowtie structure due to electron beam exposure[7]. (c) HAADF-STEM image of the TO bowtie cavity at 90° tilt. (d) EDS intensity maps of Si and O from the marked region in (c). The (projected) thickness of the surface oxide is measured to be approximately 7 nm. (e) HAADF-STEM image of the bowtie region with the sample tilted to the Si[001] zone-axis (plan-view orientation). (f) Higher-magnification HAADF-STEM image of the bowtie bridge, showing a contrast change indicated with a white arrow, which can be attributed to surface roughness and/or presence of the oxide layer. Due to minute mechanical vibrations perpendicular to the waveguide axis, only lattice planes oriented perpendicular to the waveguide axis could be observed in the power spectrum of the image in (f), shown as an inset. The spots highlighted in the power spectrum correspond to Si{220} planes. The spots were masked (indicated by orange circles), and an inverse fast Fourier transform (FFT) of the masked power spectrum yielded the image in (g), revealing a crystalline silicon bridge thickness of approximately 7 nm, which is in good agreement with the EDS measurements in (b).

S5. Simulations

Quasinormal mode calculations

For the numerical calculations, we used finite elements as implemented in Comsol Multiphysics (version: 6.1) and set up the geometry based on a trace of the cavity outline as obtained from the HAADF-STEM image of the structure at 0° and the known thickness of the silicon layer of 220 nm. This procedure comes with a certain ambiguity in the threshold, which is further complicated by native oxide with a thickness in the order of 7 nm-10 nm on all surfaces (estimated based on the EDS measurements in Figure S3). The central bowtie region is particularly sensitive to perturbations owing to the tight field confinement of the MOI,[8] and we modeled this by explicitly including a 10 nm layer of SiO_2 around both central air holes. In order to account for the oxide layer on the remaining surfaces in a simple manner, we introduced a 3 nm offset which, together with the SiO_2 in the center, serves to shift the resonance of the MOI to 0.815 eV within the narrow range of measured frequencies (cf. Figure S1). We note that this resonance frequency is very sensitive to details of the geometry, especially around the central region, so that even relatively small changes in the outline can shift it several line widths. The stated numbers represent the best estimate and associated conservative numerical error assuming the geometry is perfectly represented by the mesh. In practice, this is of little concern since the experimental uncertainty in the EELS signals is much larger than the estimated uncertainty in the numerical calculations.

EELS calculations

Electron energy-loss has been investigated theoretically with several different methods in both frequency domain[9,10] and time domain.[11] In essence, these methods all consider the field generated by a current due to an electron moving in the z-direction with constant velocity v_z and calculate the change in energy due to work done on this electron by the generated field.[9] As a result of the local electromagnetic response of the cavity, the work depends on the in-plane position of the electron beam, denoted by $\mathbf{R}_{||}$. As detailed in the literature,[9,12] the energy-loss can be written as an integral over angular frequencies as:

$$\Delta u(\mathbf{R}_{||}) = \int_0^{\infty} \hbar\omega \Delta u(\mathbf{R}_{||}, \omega) \mathrm{d}\omega \qquad \textbf{Eqn. S1}$$

in which $\hbar$ is the reduced Planck constant and the spectral energy-loss distribution, $\Delta u(\omega)$, can be written in terms of integrals along the electron trajectories as:

$$\Delta u(\mathbf{R}_{||}, \omega) = \mathrm{Im}\left\{\frac{\mathrm{e}^2\mu_0}{\pi\hbar}\int_{-\infty}^{\infty}\int_{-\infty}^{\infty} G_{zz}(\mathbf{R}_{||}, z, \mathbf{R}_{||}, z', \omega)\mathrm{e}^{\frac{-\mathrm{i}\omega(z-z')}{v_z}}\mathrm{d}z\mathrm{d}z'\right\} \qquad \textbf{Eqn. S2}$$

where e and μ_0 denote the electron charge and the vacuum permeability, respectively, and $G_{zz}(\mathbf{R}_{||},z,\mathbf{R}_{||},z',\omega)$ is the zz-component of the electric field Green tensor evaluated at $\mathbf{r} = (\mathbf{R}_{||},z)$ and $\mathbf{r}' = (\mathbf{R}_{||},z')$. In order to connect the energy-loss to the resonant fields, we followed an approach presented in the literature[10] and made use of the fact that we can expand the electric field Green tensor in terms of the QNMs, $\mathbf{f}_n$, as:[13]

$$\mathbf{G}(\mathbf{r}, \mathbf{r}', \omega) = \frac{\mathrm{c}^2}{2\omega}\sum_n \frac{\tilde{\mathbf{f}}_n(\mathbf{r}) - \tilde{\mathbf{f}}_n(\mathbf{r}')}{\tilde{\omega}_n - \omega} \qquad \textbf{Eqn. S3}$$

where c is the speed of light and the sum runs of all properly normalized QNMs of the structure. In practice, we limited the sum to the seven modes of interest, which provides a good approximation in the frequency range of interest.

S6. EELS data processing

Extracting deconvolved spectrum images with conversion to EEL probability density

The spectrum images acquired in the Gatan File Format (dm4) were processed using Hyperspy (version: 2.1.1)[14] according to the following procedure:

i. ***Spectral alignment***: The spectrum images were aligned by shifting each spectrum with sub-pixel precision so that its corresponding zero-loss peak (ZLP) is centered at 0.0 eV energy loss.

ii. ***Spatial binning***: The spectrum images were spatially binned by summing the spectra of neighboring pixels, in order to increase the signal-to-noise ratio, while sacrificing spatial resolution. A binning of 16 × 16 pixels was applied to the spectrum images for obtaining the normalized EELS probability maps. Due to the better signal-to-noise ratio in vacuum, the spatial binning of spectrum images could be reduced to 8 × 8 pixels for the normalized EELS probability maps of the vacuum regions. For the bowtie and vacuum region spectra, the high-magnification spectrum images were binned by 48 × 48 pixels.

iii. ***Richardson-Lucy (RL) deconvolution***: A RL deconvolution was applied to the spectrum images using a reference ZLP as the point spread function. The reference ZLP was recorded in vacuum and its left-hand side tail was fitted with a power-law function and reflected onto the right-hand side tail (see Figure S4). This was done to avoid unnecessary noise amplification as the result of the deconvolution operation. The same point spread function was used for all data sets, assuming no significant alterations for the conducted measurements. The RL deconvolution was limited to 20 iterations to avoid the appearance of artifacts (see Figure S4).

iv. ***Normalization***: Each spectrum was normalized with respect to its total electron counts, excluding the bulk losses in silicon, as described elsewhere.[15] In particular, this was done by obtaining the summed intensity of the ZLP within the energy range -1.12 eV-1.12 eV, as an estimate for elastic scattering plus optical losses. The normalization was done by dividing each spectrum by its corresponding summed intensity of the ZLP (see Figure S5 for a summary of these steps) and multiplied by 100 to obtain EELS probability per incoming electron in percentages (% / e^-). The EEL probability densities with the unit / eV were obtained by dividing the normalized EEL spectra by the channel dispersion value (0.01 eV).

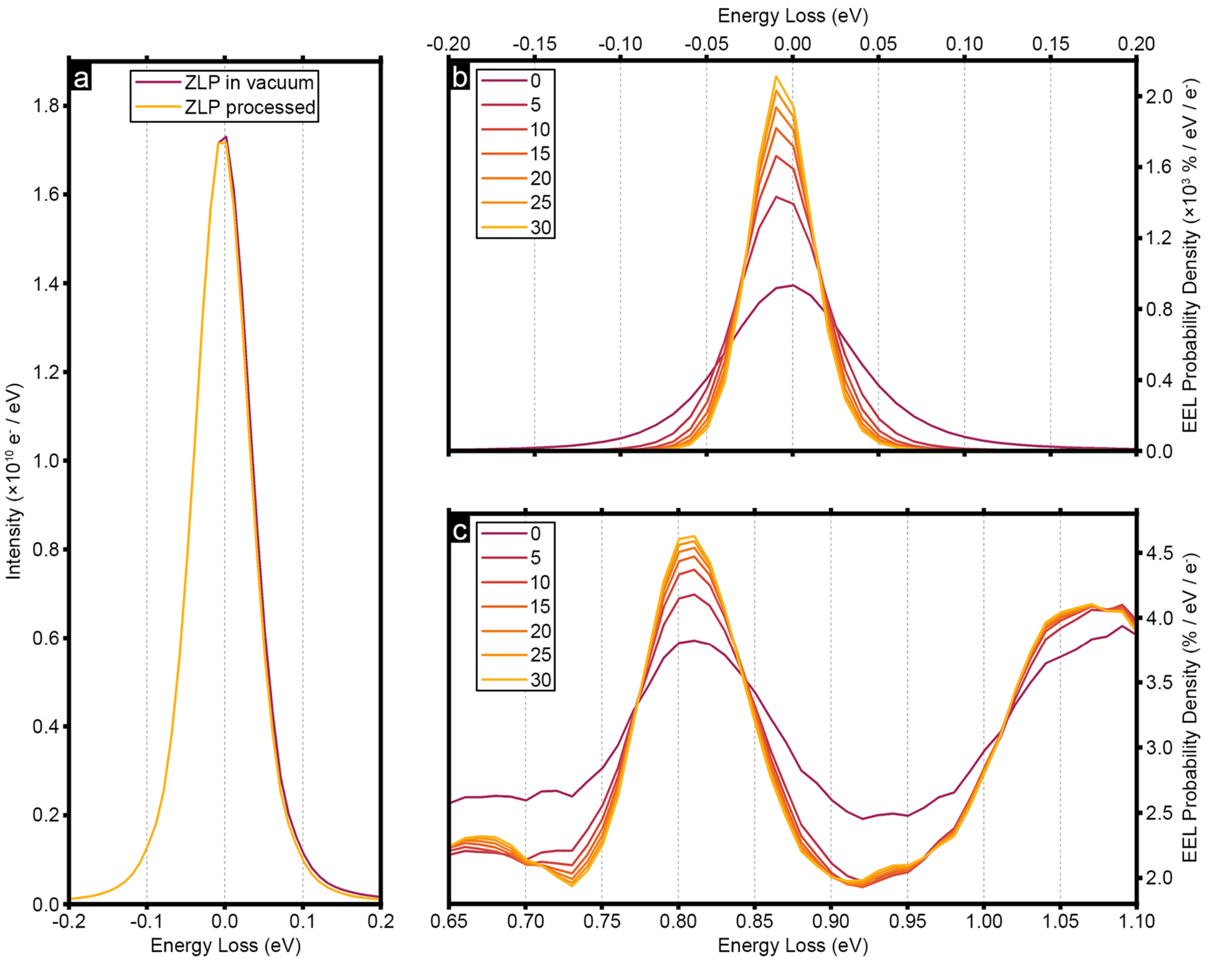

Figure S4: (a) The ZLP extracted from the vacuum region (dark magenta curve) shows slight asymmetry due to energy losses caused by the proximity of the nanophotonic structure. Therefore, the ZLP, acting as the point spread function for the subsequent RL deconvolution, was constructed by fitting the gain-side of the ZLP with a power law function and reflecting it to obtain the tail to the loss-side (yellow curve). (b) Normalized spectra after RL deconvolution using various number of iterations. (c) The same spectra plotted with a focus on the signal from the MOI, showing the evolution of the signal with increasing number of iterations. Since little improvement is observed for iterations > 20, the number of iterations for the RL deconvolution was set to 20 throughout the manuscript to avoid introducing artifacts.

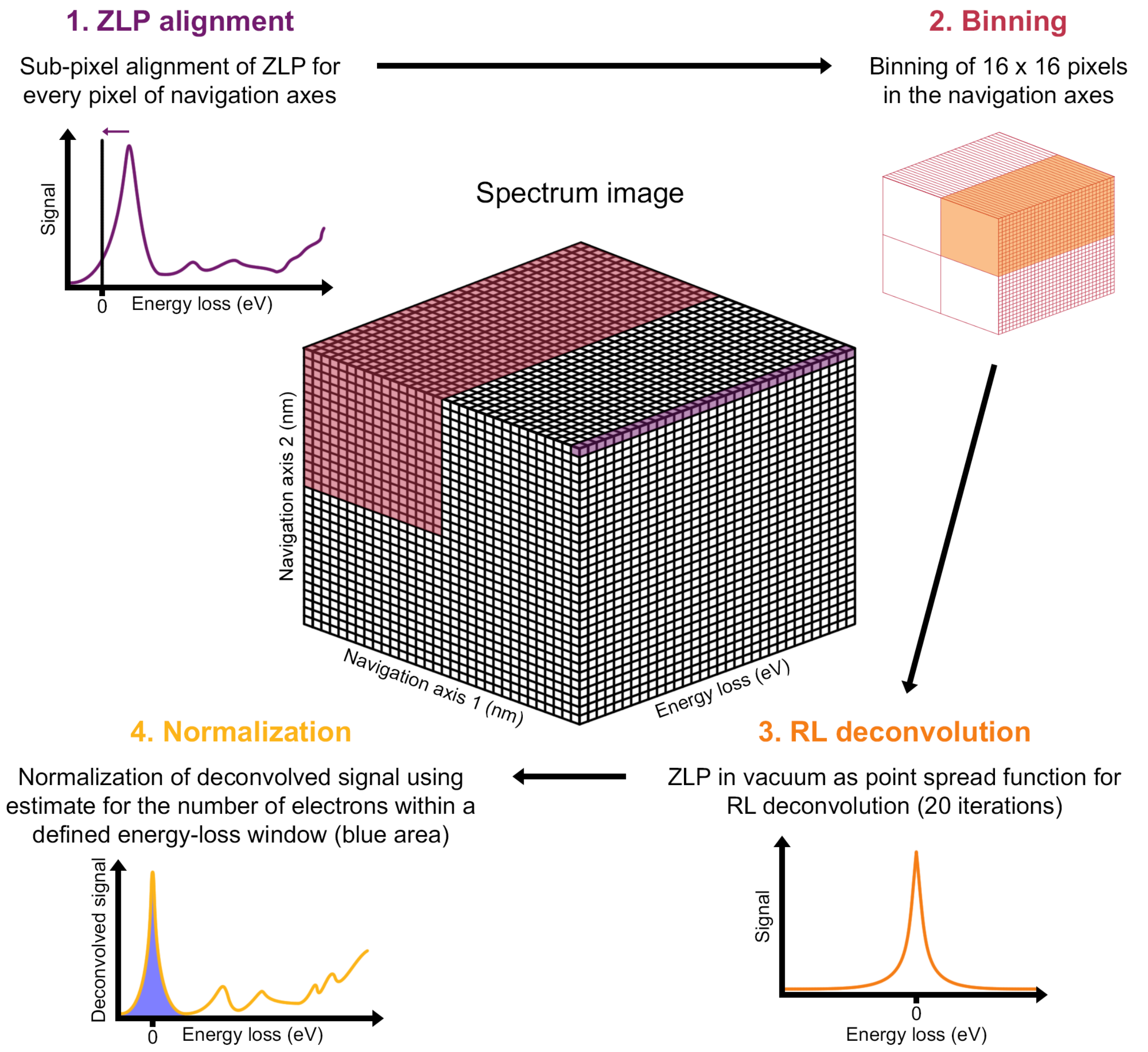

Figure S5: Schematic presentation of the spectrum image processing procedure from steps 1-4 up to the normalization with the estimate for the number of electrons within a defined energy loss window.

Preparation of EEL probability maps and line profiles by non-negative matrix factorization

The EEL probability maps were prepared by conducting non-negative matrix factorization (NMF) on the deconvolved and normalized EEL probability density spectra.[16,17] The NMF algorithm returns two matrices (both non-negative); one containing different components (see Figure S6) and the other weighting factors, which, when multiplied, reproduce the EEL spectrum image. To do this, the spectrum images acquired at 40°-90° tilt range were combined to form a four-dimensional (4D) data set (dimensions: *x*-position, *y*-position, energy loss, and tilt). The same procedure was applied to the datasets with low-magnification (whole structure) and high-magnification (bowtie region). The NMF decomposition was performed for the fixed energy range of 0.12 eV-1.5 eV and a set number of components.

For the low-magnification dataset, 28 components were identified as ideal to separate the expected modes from each other, while 32 components were chosen for the high-magnification dataset. Subsequently, the component (EEL probability spectrum) was multiplied by the corresponding weighting matrix, yielding a 4D dataset that could be associated with a specific mode. The EEL probability in the energy range 0.12 eV-1.5 eV was summed, reducing the 4D to a three-dimensional (3D) dataset. Finally, normalized EEL probability maps associated with specific sample tilts were extracted. For the vacuum region at 90° sample tilt, 34 components were chosen for the low-magnification dataset, while 30 components were chosen for the high-magnification dataset.

The value of a specific EELS map's pixel reveals the probability of an electron losing energy due to the interaction with the mode at a specific sample position. As already mentioned above, the plotted quantity is expressed in terms of % / e^-. The EEL probability maps are plotted with different boundaries for the intensity scale to enable comparisons (see Table S1 for details). The quality of the NMF approach was evaluated by comparison with the experimental bowtie EEL spectra at two different sample tilts (see Figure S7).

The experimental line profile shown in Figure 4d was prepared by averaging three neighboring pixels of the normalized EEL probability map associated with the MOI at 50° sample tilt along the direction perpendicular to the purple arrow in Figure 4b.

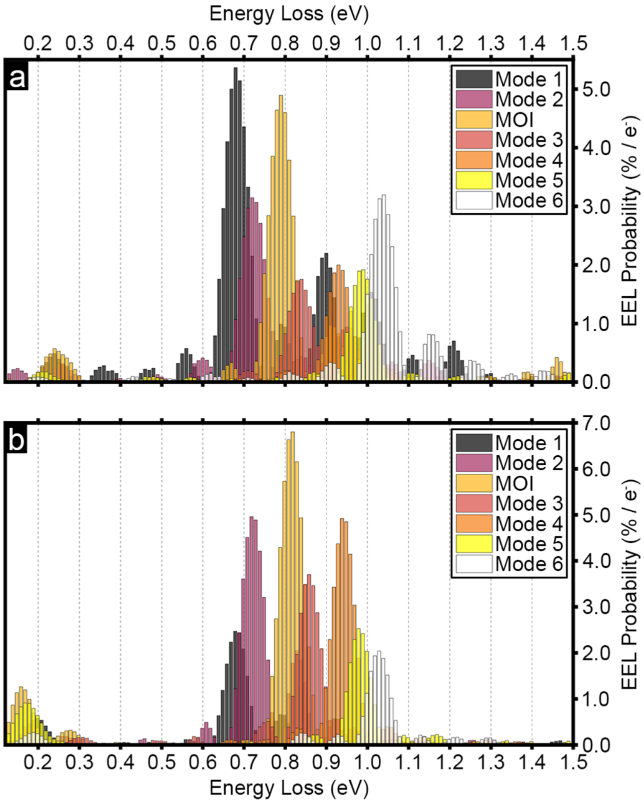

Figure S6: NMF applied to the spectrum images for (a) low- (whole structure) and (b) high-magnification (bowtie region) datasets. Note that for each dataset only the spectrum images in the tilt range 40°-90° were included in creating a 4D dataset. The EEL probability spectra in (a) and (b) reveal the extracted components that can be associated with specific modes. As described above, multiplying the weighting matrix with its component yields a set of spectrum images associated with a specific mode at different tilts. The obtained spectrum images can be used to extract individual normalized EEL probability maps of specific modes at specific tilts by summing the EEL probabilities of each energy bin in the observed energy range.

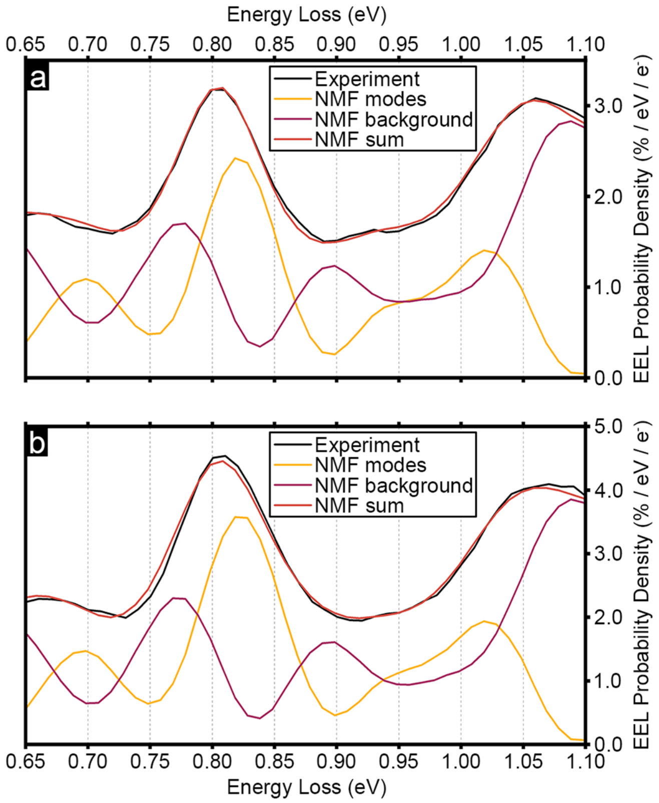

Figure S7: The bowtie EEL spectra from the TO bowtie cavity structure at (a) 50° and (b) 60° tilt (in black, see also Figure 2 of main manuscript) after binning and RL deconvolution. The spectra shown in yellow represent sums of the seven modes extracted by NMF. The purple spectra are the sums of the remaining components given by the NMF (25 components), labelled as the background. The red spectra show the sum of all components given by NMF, which, as expected, reproduce the experimental data. The backgrounds reveal features that might correspond to additional modes contributing to the overall signal, or limitations of NMF to fully separate the modes.

S7. Comparison of experimental and simulated EELS maps

A comparison between NMF, direct integration, and simulations is shown for the different modes in vacuum (90° sample tilt, Figures S8-9) and the whole region (40°-80° sample tilts, Figures S10-S13). EEL probability maps obtained without applying the NMF algorithm were prepared by plotting the EEL probability spectra of a specific 0.01 eV-wide energy bin for each mode.

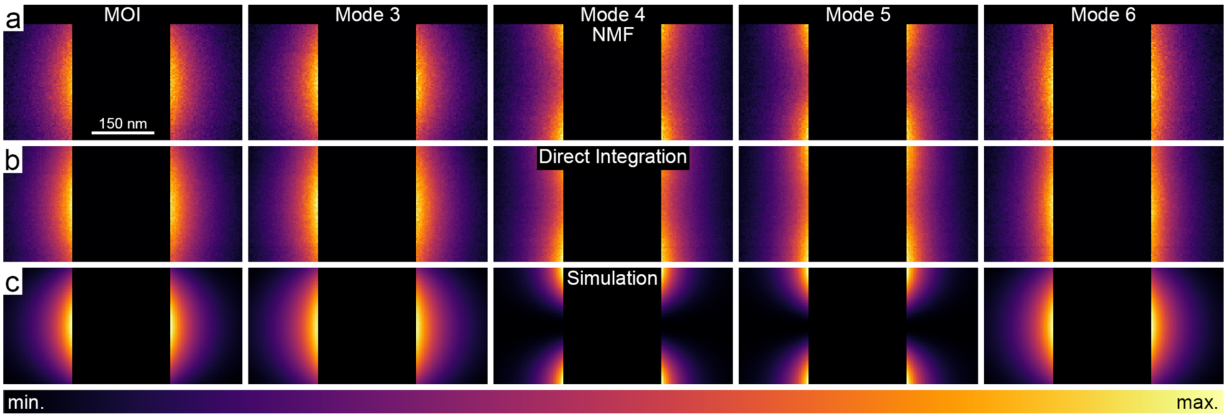

Figure S8: The EEL probability maps of the bowtie region of the TO bowtie cavity structure (pixel size: approximately 4 nm × 4 nm, sample tilt: 90°) are presented for the experimental dataset processed by the (a) NMF and (b) direct integration approaches. The experimental EEL probability maps are compared to simulations (same pixel size) in (c). The pixels that are associated with electron trajectories directly through the silicon structure were masked out and set to 0 % / e^-. Each EEL probability map associated with a specific mode and approach was plotted using an individual EEL probability scaling. For each map, the maximum and minimum EEL probability values found in the map were used as bounds of the scaling. Occasionally, small negative values appeared in the maps. In that case, the lower scaling bound was set to 0 % / e^-.

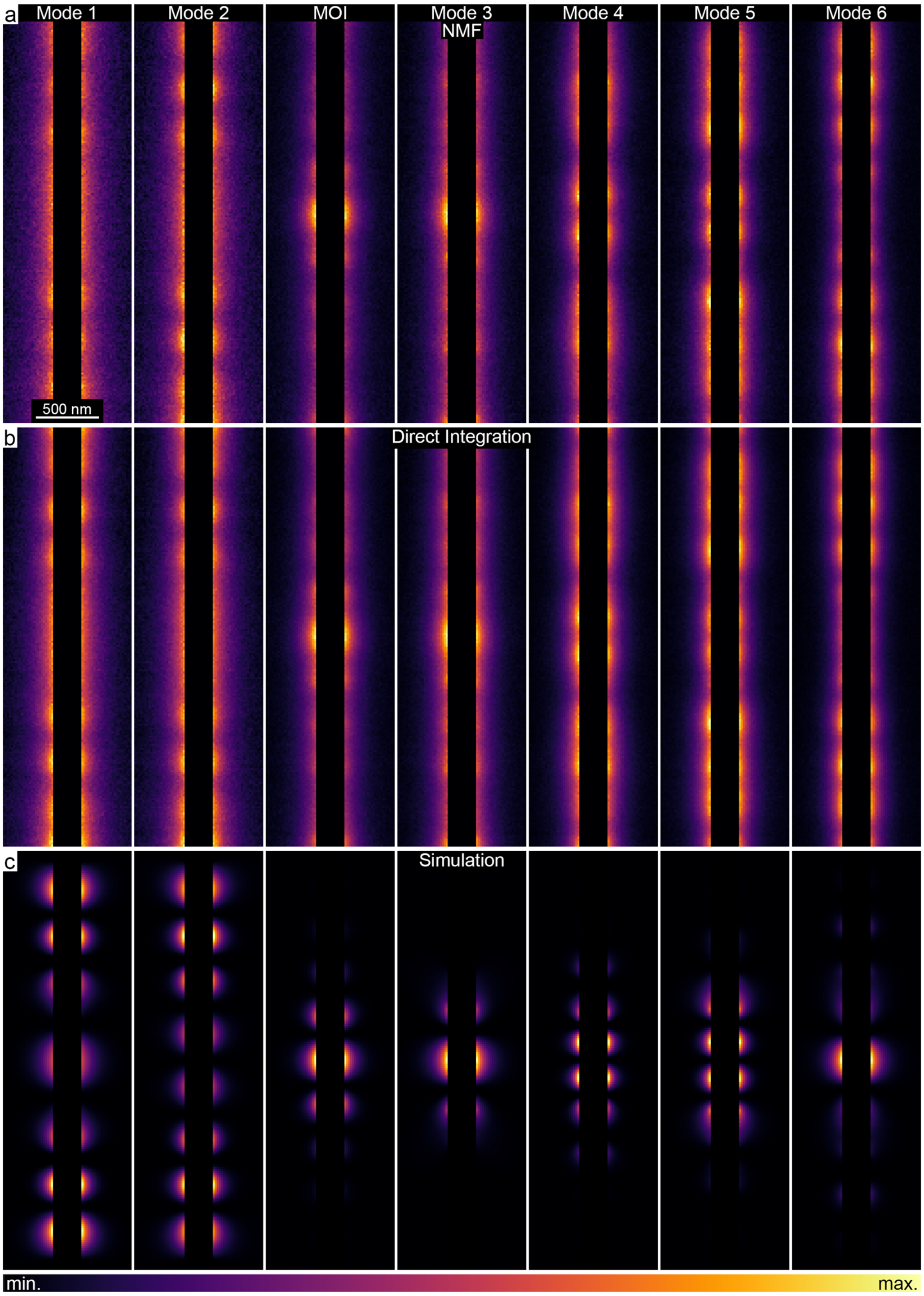

Figure S9: EEL probability maps of the whole TO bowtie cavity structure (pixel size: approximately 16 nm × 16 nm, sample tilt: 90°) presented for the experimental dataset processed by the (a) NMF and (b) direct integration approaches. The experimental EEL probability maps are compared to simulations (same pixel size) in (c). The pixels that are associated with electron trajectories directly through the silicon structure were masked out and set to 0. Each EEL probability map associated with a specific mode and approach was plotted using an individual EEL probability scaling. For each map, the maximum and minimum EEL probability values found in the map were used as bounds of the scaling. Besides Mode 6, there is a good agreement between the mode profiles extracted from the experimental dataset and the simulations.

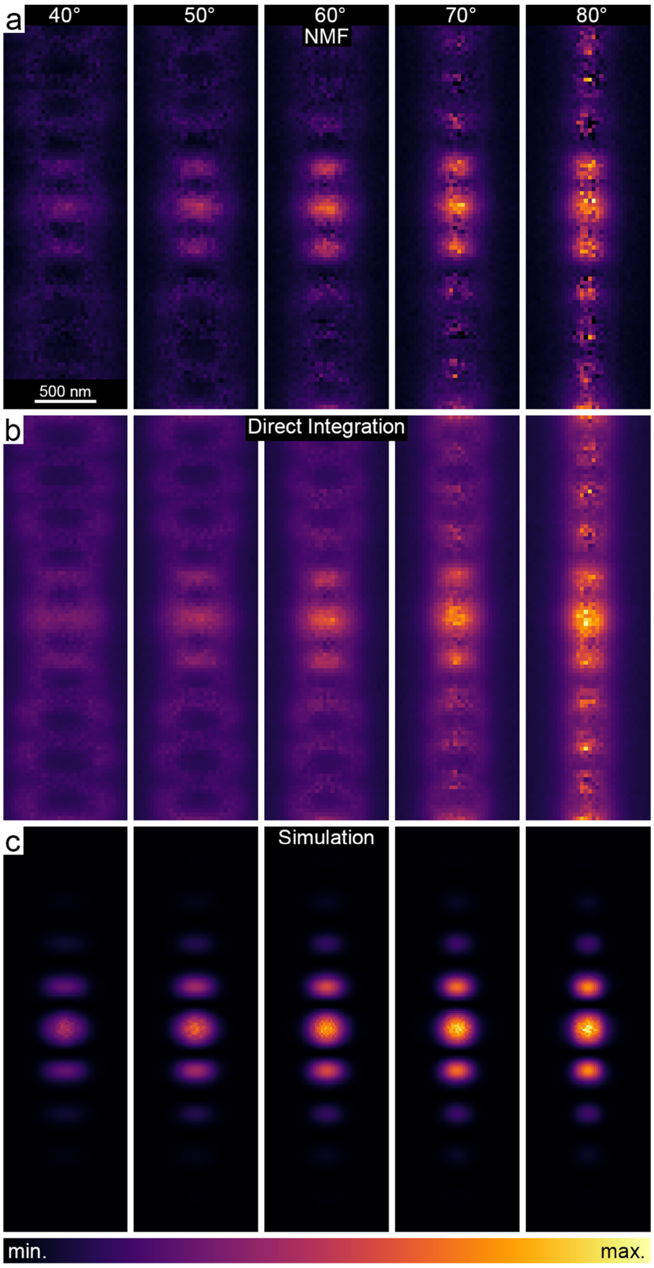

Figure S10: EEL probability maps of the whole TO bowtie cavity structure, representing a tilt series of the MOI extracted by the (a) NMF and (b) direct integration approaches (pixel size: approximately 32 nm × approximately 32 nm) in the tilt range 40°-80°, reveal an excellent agreement with the simulations (pixel size: approximately 16 nm × 16 nm) in (c). The EEL probability maps of each tilt series were plotted with the same EEL probability scaling, with the upper bound set to the maximum value in the EEL probability map at 80° sample tilt and the lower bound set to 0 % / e^-.

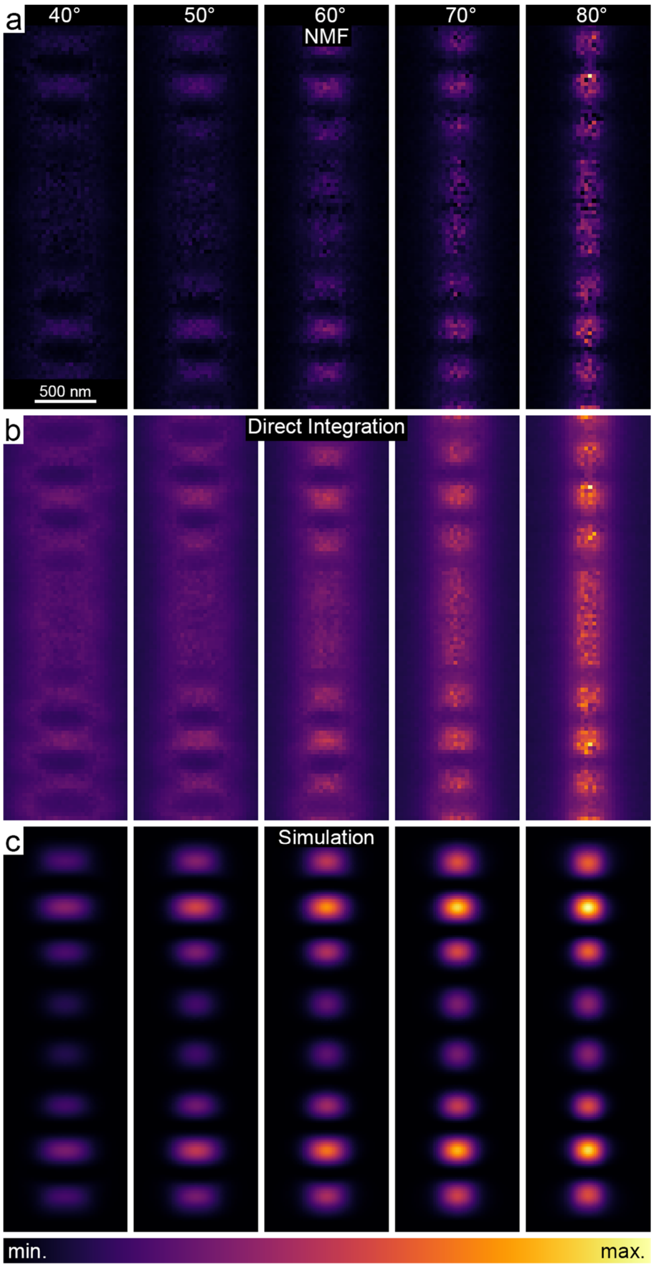

Figure S11: EEL probability maps of the whole TO bowtie cavity structure, representing a tilt series of the Mode 2 extracted by the (a) NMF and (b) direct integration approaches (pixel size: approximately 32 nm × 32 nm) in the tilt range 40°-80°, reveal an excellent agreement with the simulations (pixel size: approximately 16 nm × 16 nm) in (c). The EEL probability maps of each tilt series are plotted with the same EEL probability scaling, with the upper bound set to the maximum of the EEL probability map at 80° sample tilt and the lower bound set to 0 % / e^-.

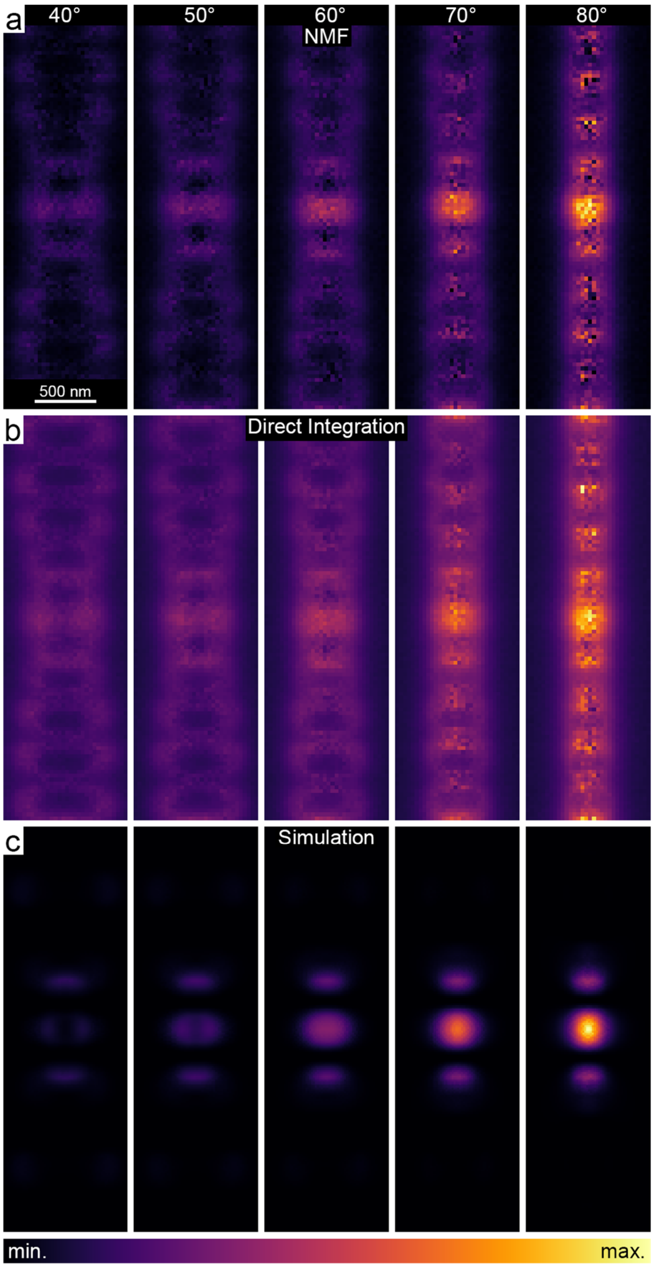

Figure S12: EEL probability maps of the whole TO bowtie cavity structure, representing a tilt series of Mode 3 extracted by the (a) NMF and (b) direct integration approaches (pixel size: approximately 32 nm × 32 nm) in the tilt range 40°-80°, revealing an excellent agreement with the simulations (pixel size: approximately 16 nm × 16 nm) in (c). The EEL probability maps of each tilt series are plotted with the same EEL probability scaling, with the upper bound set to the maximum of the EEL probability map at 80° sample tilt and the lower bound set to 0 % / e^-.

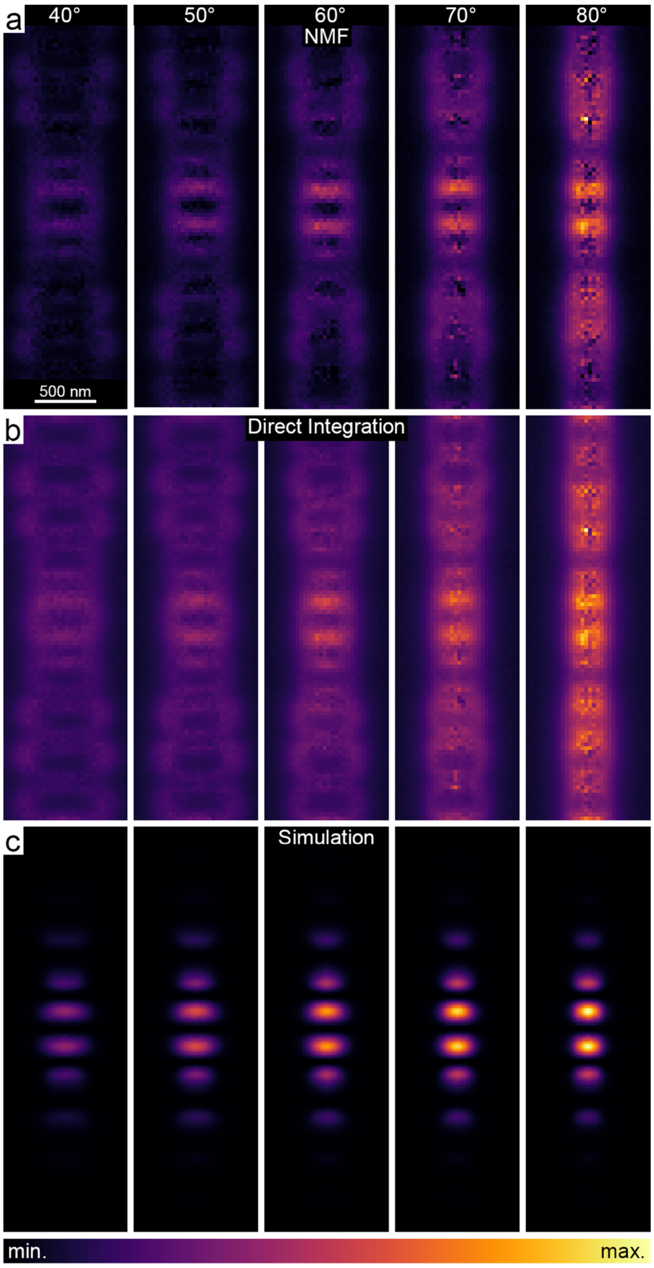

Figure S13: EEL probability maps of the whole TO bowtie cavity structure, representing a tilt series of Mode 4 extracted by the (a) NMF and (b) direct integration approaches (pixel size: approximately 32 nm × 32 nm) in the tilt range 40°-80°, revealing an excellent agreement with the simulations (pixel size: approximately 16 nm × 16 nm) in (c). The EEL probability maps of each tilt series are plotted with the same EEL probability scaling, with the upper bound set to the maximum of the EEL probability map at 80° sample tilt and the lower bound set to 0 % / e^-.

S8. Tomography

Tomographic reconstruction using HAADF-STEM images

HAADF-STEM images of the TO bowtie cavity structure recorded at different tilt angles were used to perform a tomographic reconstruction (see Figure S14). The structure was titled around its length over the range 0°-90° with 10° intervals. Two clone structures were used, one for the images in the tilt range 30°-90° and the other for those in the tilt range 0°-20° (see Figure S2g-h). First, a binning of 2 × 2 pixels was applied to the images. Subsequently, the images were normalized individually by the maximum intensity of the respective image and rotated by 90° to align the rotation axis horizontally (requirement of the used software).

The symmetry of the TO bowtie cavity structure (point group: D_{2h} (Schönflies notation)) allowed for estimating the HAADF-STEM images over the 90°-360° tilt range. Therefore, the tilt range 100°-180° was obtained by mirroring the processed HAADF-STEM images of the tilt range 80°-0°. The processed HAADF-STEM images of the tilt range 10°-170° were copied to obtain the tilt range 190°-350°. The images were processed using Hyperspy (version: 2.1.1)[14] and converted from float64 to float32 type, allowing for uploading the whole dataset to Tomviz (version: 1.10.0).[18] In Tomviz, various pre-processing steps were applied, including background subtraction, setting negative voxels to zero, normalizing the average image intensity, translation alignment, and cropping the dataset. Finally, the total variation (TV) minimization algorithm[19,20] was performed with four iterations to obtain a 3D reconstruction of the TO bowtie cavity structure.

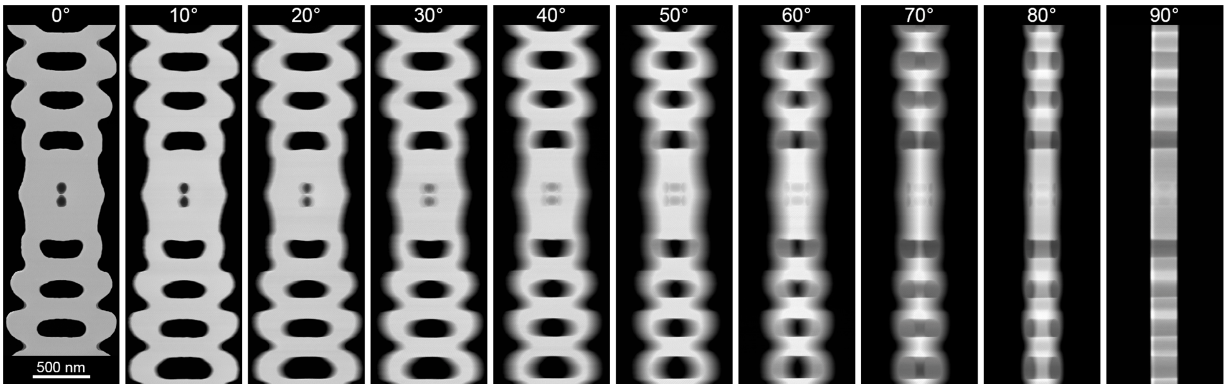

Figure S14: HAADF-STEM images of the TO bowtie cavity structure at tilts in the range 0°-90°.

Tomographic reconstruction of optical modes from experimental EEL probability maps

A similar approach as described above was chosen for reconstructing the 3D profiles of the optical modes of the TO bowtie cavity structure. In this case, only the EELS data derived for the tilt range 40°-80° and their symmetry equivalent tilt images were used for the reconstruction in order to exclude images with insufficient signal-to-noise from the reconstructions, as noted in the main manuscript. A tomographic reconstruction recovers the 3D profile of an entity from a finite number of projected two-dimensional (2D) images. Working in the coordinate system of Figure 1, and assuming the normalized QNM to be approximately real and x-polarized, we can write the nth term of the spectral energy loss distribution as:

$$\Delta u_n(\mathbf{R}_\theta, \omega) = \frac{\mathrm{e}^2 \mathrm{c}^2 \mu_0}{2\pi\hbar} \cos^2(90° - \theta) \, \frac{\gamma_n/\omega}{(\tilde{\omega}_n - \omega)^2 + \gamma_n^2} \times \left| \int_{-\infty}^{+\infty} \tilde{\mathbf{f}}_n(\mathbf{R}_\theta, z') \, \mathrm{e}^{\frac{-\mathrm{i}\omega z'}{v_z}} \mathrm{d}z' \right|^2 \qquad \textbf{Eqn. S4}$$

where θ is the sample tilt angle. This provides the relationship between the EEL probability maps and the x-polarized optical modes and shows that, assuming a negligible exponential term in Eqn. S4 and negligible mode overlap, the EELS probability maps obtained at the real part of the relevant resonance frequencies become proportional to $\cos^2(90° - \theta) \times | \int_{-\infty}^{+\infty} \tilde{\mathbf{f}}_n(\mathbf{R}_\theta, z') \mathrm{d}z' |^2$, with the term $| \int_{-\infty}^{+\infty} \tilde{\mathbf{f}}_n(\mathbf{R}_\theta, z') \mathrm{d}z' |^2$ signifying the modulus square of the mode profile.

Therefore, the square root of the EEL probability maps divided by cos(90°-θ) were calculated for each map prior to applying the tomographic reconstruction procedure. Subsequently, the residual background present in the processed EEL probability maps was removed following the procedure exemplarily highlighted in Figure S15. In Tomviz, a translation alignment of the stack was performed and a Gaussian smoothing with σ = 1.0 was applied to the individual maps. Finally, the TV minimization reconstruction algorithm was conducted with five iterations to obtain the 3D reconstruction of specific modes.

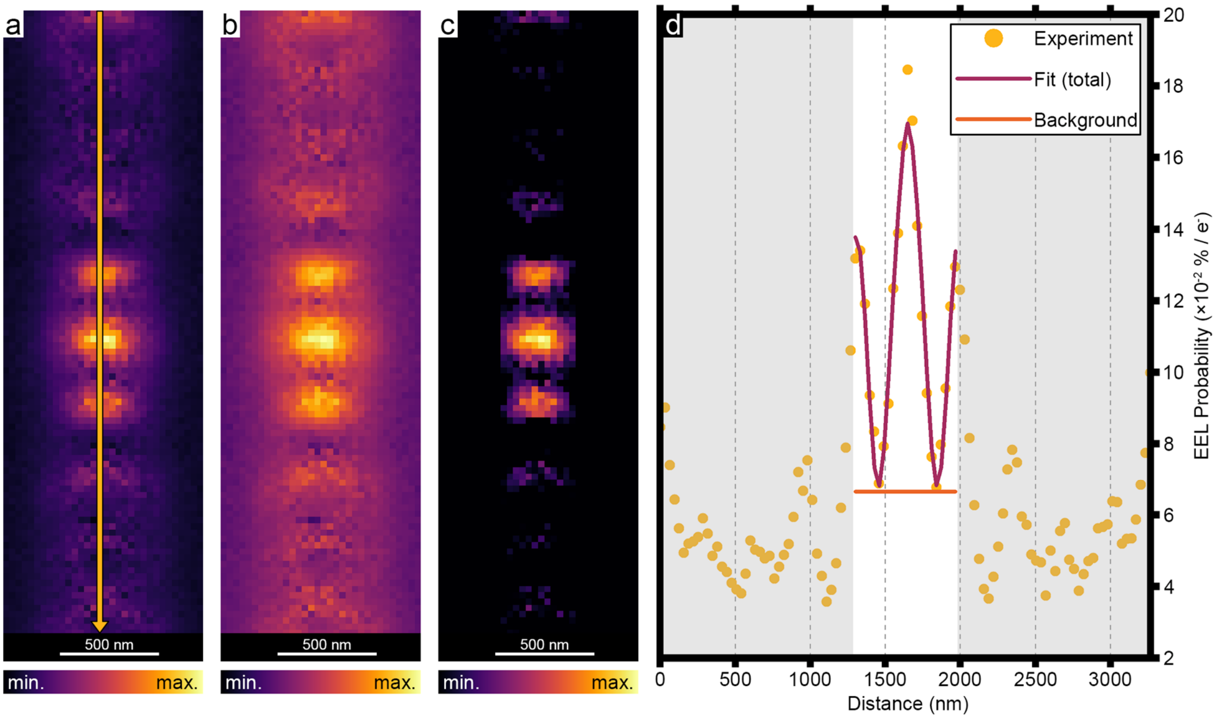

Figure S15: The procedure for processing EEL probability maps prior to the reconstruction procedure is exemplarily explained. (a) The EEL probability map at 60° sample tilt was processed by taking the square root of the EEL probability and dividing by cos(30°) for each pixel. (b) The described procedure yielded a map that is proportional to the projected profile of the normalized electric field strength along the polarization-direction. (c) After removing the background, the obtained map can be used for the reconstruction procedure. (d) The background was removed by plotting the line profile from the EEL probability map in (a) and fitting it with the line profile extracted from the corresponding simulated EEL probability map (see Figure S10c) plus a constant value. For preparing the experimental line profile, 15 neighboring pixels were averaged along the direction perpendicular to the orange arrow in (a). The same procedure was used for preparing the simulated line profile. The fitted constant value was used to estimate the residual background present in the experimental EEL probability map and was subsequently subtracted from the map. This procedure was performed on all experimental EEL probability maps before reconstructing the 3D profiles of the optical modes.

Tomographic reconstruction of optical modes from simulated EELS maps

In the case of the theoretical data, the simulated EEL probability maps in the tilt range 40°-80° and the ones deduced from them due to symmetry were used for the reconstruction. Any small negative values were set to 0 % / e^- prior to the processing in Tomviz. Analogous to the above, the EEL probabilities of the maps were converted to a quantity direct proportional to the normalized electric field strength along the *x*-direction and rotated. In Tomviz, the same Gaussian smoothing procedure with $\sigma = 1.0$ was applied to the individual maps for a better comparison between the experimental and simulated datasets. Subsequently, a TV minimization procedure with five iterations was performed to obtain a 3D reconstruction of specific modes. Additional steps, as outlined above, were not required for a 3D reconstruction using the simulated dataset.

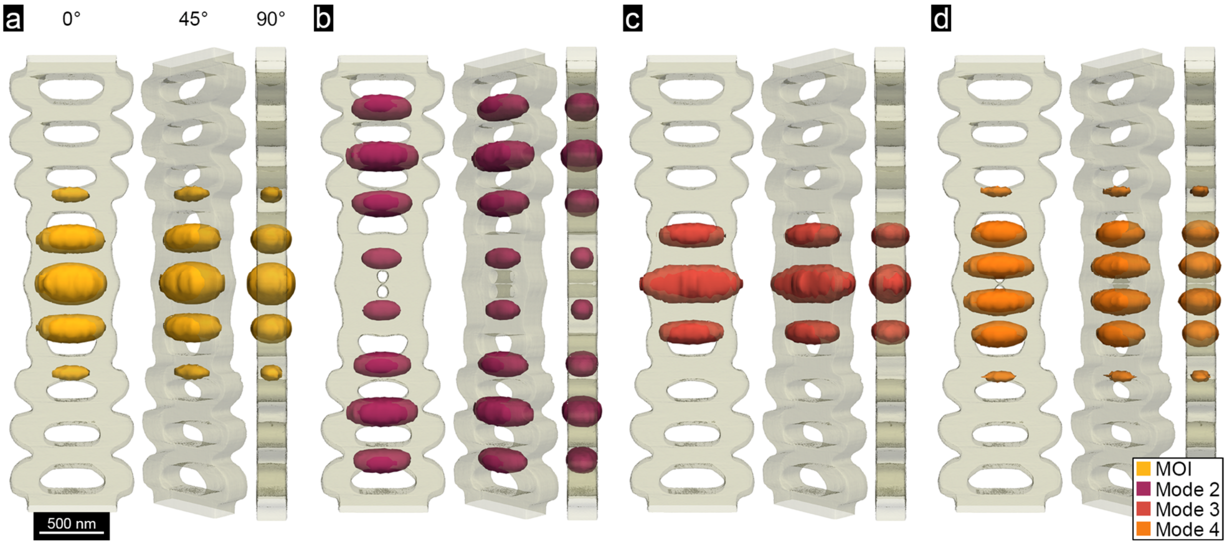

Figure S16: Isosurface renderings of the 3D profiles at 35 % of the MOI's $|\mathbf{E}_x|_{max}$ value obtained from tomographic reconstructions of the simulated EEL probability maps for the (a) MOI, (b) Mode 2, (c) Mode 3, and (d) Mode 4. The isosurfaces obtained from simulated EEL probability maps show an excellent agreement with the ones obtained from the experimental EEL probability maps presented in Figure 5 of the main manuscript.

Table S1: Pixel sizes and boundaries of the intensity bars of the presented normalized EEL probability maps in the main manuscript and supporting information.

Figure	Approximate side length of square pixel (nm)	Tilt (°)	Mode	Approach	Min. EEL probability (% / e^-)	Max. EEL probability (% / e^-)
3a	15.9	90	MOI	NMF	0.0187	0.2100
3a	15.9	90	1	NMF	0.0326	0.1322
3a	15.9	90	2	NMF	0.0284	0.1281
3a	15.9	90	3	NMF	0.0201	0.1876
3a	15.9	90	4	NMF	0.0157	0.2107
3a	15.9	90	5	NMF	0.0133	0.1955
3a	15.9	90	6	NMF	0.0152	0.2270
3a	15.9	90	MOI	Simulation	0.0000	0.0752
3a	15.9	90	1	Simulation	0.0000	0.0494
3a	15.9	90	2	Simulation	0.0000	0.0468
3a	15.9	90	3	Simulation	0.0000	0.1364
3a	15.9	90	4	Simulation	0.0000	0.0413
3a	15.9	90	5	Simulation	0.0000	0.0704
3a	15.9	90	6	Simulation	0.0000	0.0770
3b	3.7	90	MOI	NMF	0.0267	0.1811
3b	3.7	90	MOI	Simulation	0.0015	0.0845
3b	3.7	90	4	NMF	0.0343	0.2252
3b	3.7	90	4	Simulation	0.0000	0.0483
4b	31.7	40-80	MOI	NMF	0.0000	0.4236
4c	5.7	40-80	MOI	Simulation	0.0000	0.1912
4e	7.3	60	MOI	NMF	0.0122	0.2853
4e	7.3	60	MOI	Simulation	0.0006	0.1590
S8a	3.7	90	MOI	NMF	0.0267	0.1811
S8a	3.7	90	3	NMF	0.0283	0.2144

S8a	3.7	90	4	NMF	0.0343	0.2252
S8a	3.7	90	5	NMF	0.0221	0.2157
S8a	3.7	90	6	NMF	0.0211	0.1552
S8b	3.7	90	MOI	Direct integration	0.0091	0.0486
S8b	3.7	90	3	Direct integration	0.0092	0.0465
S8b	3.7	90	4	Direct integration	0.0080	0.0496
S8b	3.7	90	5	Direct integration	0.0069	0.0451
S8b	3.7	90	6	Direct integration	0.0055	0.0327
S8c	3.7	90	MOI	Simulation	0.0015	0.0845
S8c	3.7	90	3	Simulation	0.0058	0.1466
S8c	3.7	90	4	Simulation	0.0000	0.0483
S8c	3.7	90	5	Simulation	0.0000	0.0774
S8c	3.7	90	6	Simulation	0.0028	0.0840
S9a	15.9	90	MOI	NMF	0.0187	0.2100
S9a	15.9	90	1	NMF	0.0326	0.1322
S9a	15.9	90	2	NMF	0.0284	0.1281
S9a	15.9	90	3	NMF	0.0201	0.1876
S9a	15.9	90	4	NMF	0.0157	0.2107
S9a	15.9	90	5	NMF	0.0133	0.1955
S9a	15.9	90	6	NMF	0.0152	0.2270
S9b	15.9	90	MOI	Direct integration	0.0058	0.0461
S9b	15.9	90	1	Direct integration	0.0073	0.0289
S9b	15.9	90	2	Direct integration	0.0072	0.0302
S9b	15.9	90	3	Direct integration	0.0055	0.0442
S9b	15.9	90	4	Direct integration	0.0049	0.0500
S9b	15.9	90	5	Direct integration	0.0043	0.0506
S9b	15.9	90	6	Direct integration	0.0042	0.0540

S9c	15.9	90	MOI	Simulation	0.0000	0.0752
S9c	15.9	90	1	Simulation	0.0000	0.0494
S9c	15.9	90	2	Simulation	0.0000	0.0468
S9c	15.9	90	3	Simulation	0.0000	0.1364
S9c	15.9	90	4	Simulation	0.0000	0.0413
S9c	15.9	90	5	Simulation	0.0000	0.0704
S9c	15.9	90	6	Simulation	0.0000	0.0770
S10a	31.7	40-80	MOI	NMF	0.0000	0.4236
S10b	31.7	40-80	MOI	Direct integration	0.0000	0.0691
S10c	15.7	40-80	MOI	Simulation	0.0000	0.1912
S11a	31.7	40-80	2	NMF	0.0000	0.6717
S11b	31.7	40-80	2	Direct integration	0.0000	0.0718
S11c	15.7	40-80	2	Simulation	0.0000	0.1319
S12a	31.7	40-80	3	NMF	0.0000	0.3711
S12b	31.7	40-80	3	Direct integration	0.0000	0.0620
S12c	15.7	40-80	3	Simulation	0.0000	0.3081
S13a	31.7	40-80	4	NMF	0.0000	0.4246
S13b	31.7	40-80	4	Direct integration	0.0000	0.0736
S13c	15.7	40-80	4	Simulation	0.0000	0.1514
S15a	31.7	60	MOI	NMF	0.0000	0.2892
S15a	31.7	60	MOI	NMF processed	0.0000 (a.u.)	0.6210 (a.u.)
S15a	31.7	60	MOI	NMF processed	0.0000 (a.u.)	0.3232 (a.u.)

References

1. Christiansen, R. E. Inverse design of optical mode converters by topology optimization: tutorial. *J. Opt.* **25**, 083501 (2023).
2. Zhou, M., Lazarov, B. S., Wang, F. & Sigmund, O. Minimum length scale in topology optimization by geometric constraints. *Comput. Methods Appl. Mech. Eng.* **293**, 266–282 (2015).
3. Li, Q., Chen, W., Liu, S. & Tong, L. Structural topology optimization considering connectivity constraint. *Struct. Multidisc. Optim.* **54**, 971–984 (2016).
4. Dong, G. *et al.* Enhancement and speed-up of carrier dynamics in a dielectric nanocavity with deep sub-wavelength confinement. *arXiv preprint arXiv:2412.08471* https://doi.org/10.48550/arXiv.2412.08471 (2024) doi:10.48550/arXiv.2412.08471.
5. Tortorelli, D. A. & Michaleris, P. Design sensitivity analysis: Overview and review. *Inverse Probl. Eng.* **1**, 71–105 (1994).
6. Babar, A. N. Fabrication and characterization of silicon photonic cavities with atomic-scale confinement. (Technical University of Denmark, 2024).
7. Hettler, S. *et al.* Carbon contamination in scanning transmission electron microscopy and its impact on phase-plate applications. *Micron* **96**, 38–47 (2017).
8. Kountouris, G., Mørk, J., Denning, E. V. & Kristensen, P. T. Modal properties of dielectric bowtie cavities with deep sub-wavelength confinement. *Opt. Express* **30**, 40367 (2022).
9. García de Abajo, F. J. & Kociak, M. Probing the Photonic Local Density of States with Electron Energy Loss Spectroscopy. *Phys. Rev. Lett.* **100**, 106804 (2008).
10. Ge, R.-C. & Hughes, S. Quasinormal mode theory and modelling of electron energy loss spectroscopy for plasmonic nanostructures. *J. Opt.* **18**, 054002 (2016).
11. Matyssek, C., Niegemann, J., Hergert, W. & Busch, K. Computing electron energy loss spectra with the Discontinuous Galerkin Time-Domain method. *Photonics Nanostruct.* **9**, 367–373 (2011).
12. García de Abajo, F. J. Optical excitations in electron microscopy. *Rev. Mod. Phys.* **82**, 209–275 (2010).
13. Kristensen, P. T., Herrmann, K., Intravaia, F. & Busch, K. Modeling electromagnetic resonators using quasinormal modes. *Adv. Opt. Photonics* **12**, 612 (2020).
14. de la Peña, F. *et al.* hyperspy/hyperspy: v2.1.1. Preprint at https://doi.org/10.5281/zenodo.12724131 (2024).
15. Le Thomas, N. *et al.* Imaging of high-Q cavity optical modes by electron energy-loss microscopy. *Phys. Rev. B* **87**, 155314 (2013).
16. Lee, D. D. & Seung, H. S. Learning the parts of objects by non-negative matrix factorization. *Nature* **401**, 788–791 (1999).

17. Nicoletti, O. *et al.* Three-dimensional imaging of localized surface plasmon resonances of metal nanoparticles. *Nature* **502**, 80–84 (2013).

18. Schwartz, J. *et al.* Real-time 3D analysis during electron tomography using tomviz. *Nat. Commun.* **13**, 4458 (2022).

19. Sidky, E. Y. & Pan, X. Image reconstruction in circular cone-beam computed tomography by constrained, total-variation minimization. *Phys. Med. Biol.* **53**, 4777–4807 (2008).

20. Sanders, T., Gelb, A., Platte, R. B., Arslan, I. & Landskron, K. Recovering fine details from under-resolved electron tomography data using higher order total variation ℓ1 regularization. *Ultramicroscopy* **174**, 97–105 (2017).
