# Supplementary figures and images for: 3D Imaging of Optical Modes in Dielectric Photonic Nanocavities with Sub-wavelength Field Confinement
Source: Nano Lett. 2025 Nov 10;25(46):16395–403. doi: 10.1021/acs.nanolett.5c04226 (PMC12636085; doi:10.1021/acs.nanolett.5c04226)

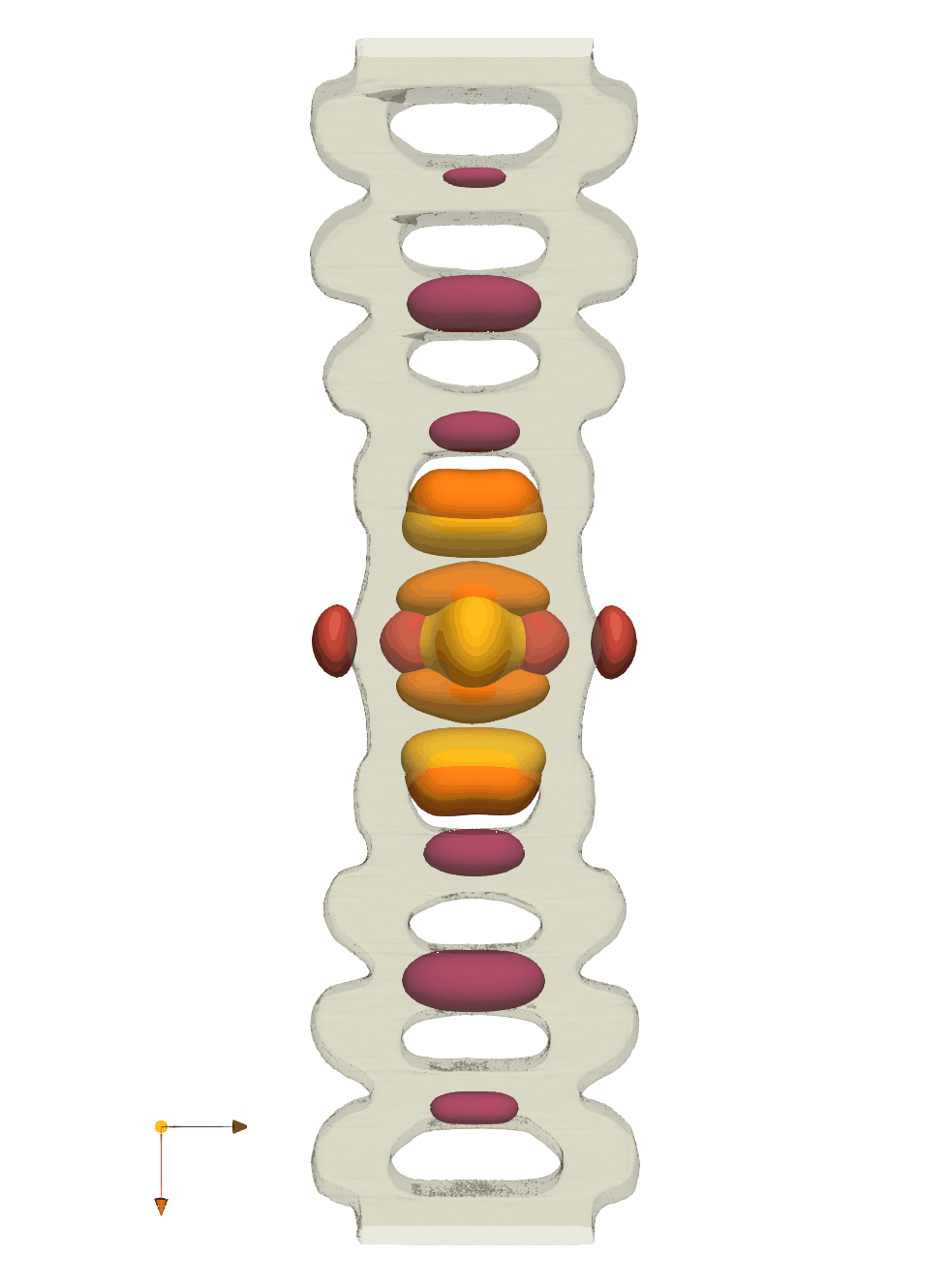

Supplement: Supplementary file 2 [file nl5c04226_si_002.gif]

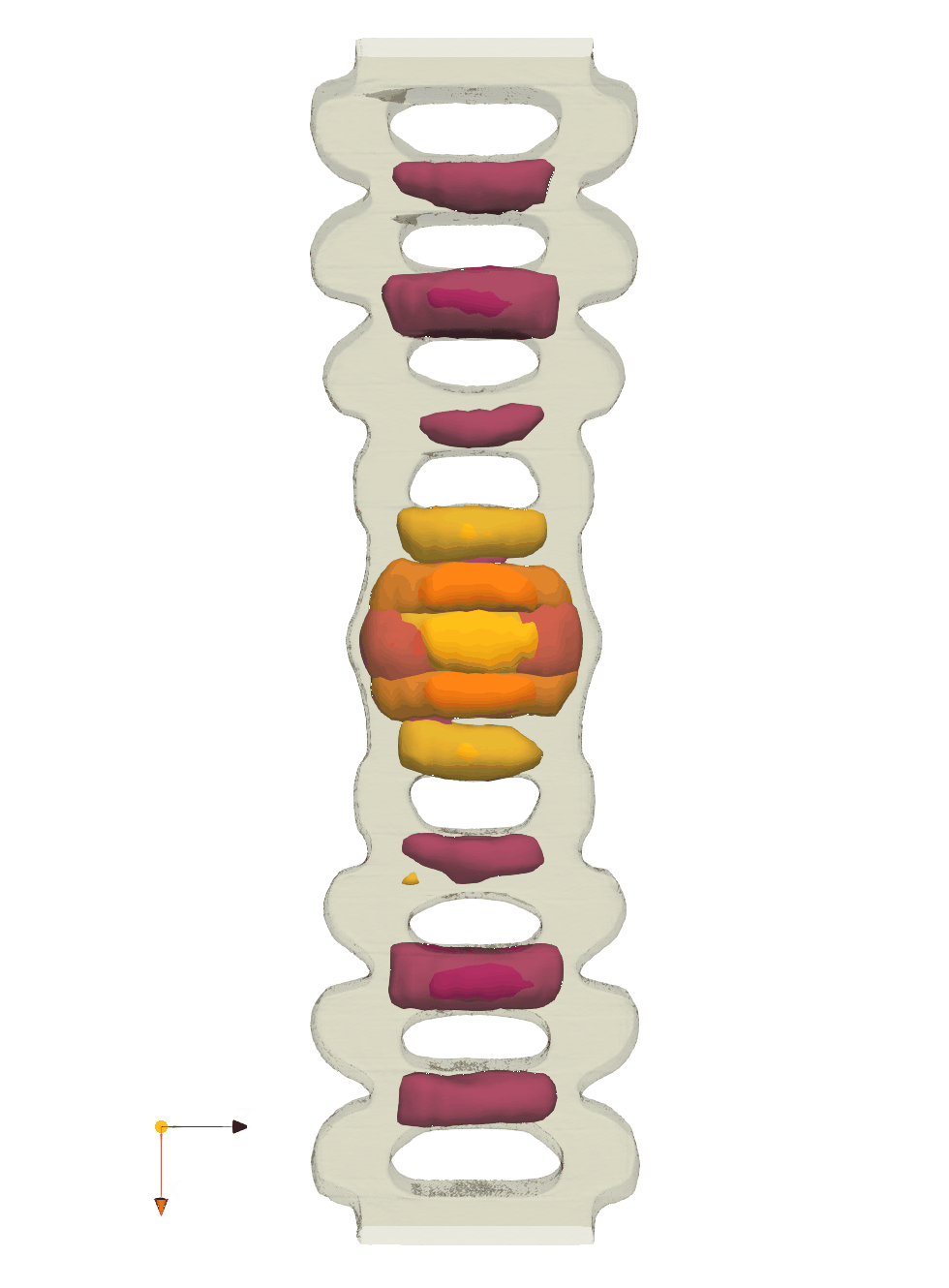

Supplement: Supplementary file 3 [file nl5c04226_si_003.gif]
